# Identification of biomarkers and the mechanisms of multiple trauma complicated with sepsis using metabolomics

**DOI:** 10.3389/fpubh.2022.923170

**Published:** 2022-08-04

**Authors:** Ke Feng, Wenjie Dai, Ling Liu, Shengming Li, Yi Gou, Zhongwei Chen, Guodong Chen, Xufeng Fu

**Affiliations:** ^1^Department of Emergency, General Hospital of Ningxia Medical University, Yinchuan, China; ^2^Key Laboratory of Fertility Preservation and Maintenance of Ministry of Education, School of Basic Medical Sciences, Ningxia Medical University, Yinchuan, China

**Keywords:** biomarkers, multiple trauma, sepsis, metabolomics, mechanisms

## Abstract

Sepsis after trauma increases the risk of mortality rate for patients in intensive care unit (ICUs). Currently, it is difficult to predict outcomes in individual patients with sepsis due to the complexity of causative pathogens and the lack of specific treatment. This study aimed to identify metabolomic biomarkers in patients with multiple trauma and those with multiple trauma accompanied with sepsis. Therefore, the metabolic profiles of healthy persons designated as normal controls (NC), multiple trauma patients (MT), and multiple trauma complicated with sepsis (MTS) (30 cases in each group) were analyzed with ultra-high performance liquid chromatography coupled with quadrupole time-of-flight mass spectrometry (UHPLC-Q-TOF/MS)-based untargeted plasma metabolomics using collected plasma samples. The differential metabolites were enriched in amino acid metabolism, lipid metabolism, glycometabolism and nucleotide metabolism. Then, nine potential biomarkers, namely, acrylic acid, 5-amino-3-oxohexanoate, 3b-hydroxy-5-cholenoic acid, cytidine, succinic acid semialdehyde, PE [P-18:1(9Z)/16:1(9Z)], sphinganine, uracil, and uridine, were found to be correlated with clinical variables and validated using receiver operating characteristic (ROC) curves. Finally, the three potential biomarkers succinic acid semialdehyde, uracil and uridine were validated and can be applied in the clinical diagnosis of multiple traumas complicated with sepsis.

## Introduction

Trauma is one of the leading causes of morbidity and mortality among all age populations worldwide (1, 2). Multiple trauma is common injury at two or more anatomical sites caused by a single consistent injury factor. Besides the health status, multiple trauma can trigger a complex cascade of posttraumatic events, including massive secretion of proinflammatory cytokines, an imbalance between the early systemic inflammatory response, later compensatory anti-inflammatory response, and even multiple organ failure, which are closely correlated with the outcomes of victims (3). Sepsis is a major cause of mortality in critically ill patients, especially patients with multiple trauma. Sepsis is a life-threatening condition caused by the body's extreme response to infection. It causes nearly six million deaths worldwide annually (4). Multiple trauma also causes sepsis. The trauma-induced sepsis is the leading cause of a high mortality rate in intensive care units (ICUs). Moreover, sepsis associated with multiple organ dysfunction syndrome is the primary cause of late posttraumatic mortality, accounting for up to 50% (5, 6). Although various advanced technologies, such as bundled early goal-directed therapy, have been used to sepsis, sepsis prognosis is still poor (7, 8). Furthermore, the high mortality associated with sepsis is partially due to the lack of an effective approach to predict sepsis outcomes. It is difficult to diagnose multiple trauma-induced sepsis because the hypermetabolic baseline and the explosive inflammatory immune response mask the clinical signs and symptoms of sepsis (9, 10). Therefore, it is necessary to determine promising biomarkers for patients with multiple trauma without sepsis to estimate the individual risk profile and prevent sepsis development.

Previous diagnostic definitions and manifestations of sepsis, including Glasgow or sequential organ failure assessment (SOFA) scores, have been performed based on sepsis 3.0 due to the substantial heterogeneity of clinical syndrome (11). The laboratory testing of sepsis is currently based on the related factors of acute immune response caused by host reactants in serum. C-reactive protein and procalcitonin have been widely used in clinics for infection diagnosis and sepsis progression prediction (12). Metabolite lactate has been standardized for indications of sepsis and septic shock. Furthermore, studies have shown that the proinflammatory cytokines interleukin-6 and tumor necrosis factor-alpha can be used as markers to diagnose sepsis (13, 14). Although patient blood culture is recommended for diagnosing the etiologic agent of sepsis, sepsis cannot be detected in most patients due to the low abundance of microorganisms in the bloodstream or because the organisms cannot be proliferated in conventional culture medium (15). However, these biomarkers are universal and non-specific in sepsis. Besides, the difficulty of early sepsis diagnosis and the limited knowledge of the molecular mechanism of sepsis development limits the timely treatment of sepsis (16). Therefore, specific biomarkers for sepsis diagnosis should be detected to help differentiate between the various factors and conditions associated with sepsis.

Omics technologies can identify biomarkers by detecting biochemical changes associated with the gene expression at the transcription and translation levels and metabolites in the overall biological state (17). Metabolomics is widely used to assess all metabolites contained in an organism. For instance, genome, transcriptome or proteome changes can be reflected in the metabolome as alterations of metabolite concentration (18). As a result, metabolomics can identify novel and potential metabolite markers and explore molecular mechanisms in various diseases, including sepsis, through blood detection. Metabolomics technology can also globally evaluate the totality of endogenous metabolites in the body of sepsis patients and reflect gene function and enzyme activity (19). However, metabolomics can be used to quantitatively distinguish patients with sepsis from healthy individuals by analyzing several low molecular weight compounds, such as amino acids, fatty acids, nucleotides and their derivatives, which are important in diagnosis and pathogenesis (20). However, clinical studies on sepsis metabonomics have not identified any specific biomarkers for multiple injuries-induced sepsis.

This study used untargeted metabolomics based on ultrahigh-performance liquid chromatography coupled with quadrupole time-of-flight mass spectrometry (UHPLC-Q-TOF/MS) to screen several metabolites in plasma samples of multiple trauma complicated with sepsis (MTS). A computational bioinformatics analysis was then used to obtain numerous significantly different metabolites. The metabolites were further analyzed based on the clinical data and characteristics of patients to obtain a set of potential metabolites that can be used in the clinical diagnosis and detection of MTS.

## Materials and methods

### Patients

This study was carried out in line with the Declaration of Helsinki and approved by the Ethics Committee of the General Hospital of Ningxia Medical University (No. 2020-34). All patients provided written informed consent. For ICU patients and those with serious multiple traumas, consents were provided by their legal guardians. Plasma samples were obtained from 30 patients with multiple trauma (MT) and 30 patients with multiple trauma complicated with sepsis (MTS) who were admitted in the outpatient room of the emergency department between 2016 and 2019. In addition, 30 samples were obtained from healthy normal individuals (NC, aged from 30 to 50) from the Healthy Examination Center of the General Hospital of Ningxia Medical University. All healthy volunteers were fully informed of the study details and agreed to participate in the investigation, and also provided written informed consent. Patients with MT or MTS were enrolled according to Sepsis-3 definition., complete basic information of patients was obtained. Plasma samples were collected within 1 h of hospitalization and before antibiotic treatment (21). Sequential Organ Failure Assessment (SOFA) score and Glasgow score were calculated to assess sepsis severity, and the scores were confirmed by two pathologists. Serum biochemical information of patients was obtained from the hospital database.

### Preparation of plasma samples and extraction of metabolites

Plasma samples were collected from NC, MT, and MTS groups (30 patients for each group). The plasma samples were stored at −80°C and thawed at 4°C before LC-MS/MS analysis. Briefly, 200 μL of the extraction solution composed of acetonitrile/methanol (1:1, v/v) and isotopically labeled internal standard was added to 50 μL of each plasma sample, and mixed by vortexing for 30 s. It was sonicated for 10 min, and incubated for 1 h at −40°C. After centrifugation at 4°C and 12,000 g for 15 min, the supernatant was collected into a fresh glass vial for subsequent analysis. To ensure credibility of analysis, a bulk quality control (QC) sample was prepared by mixing equal volume aliquots and used for monitoring LC/MS response and calibrating data.

### LC-MS/MS analysis

Untargeted metabolite profile of plasma samples was performed using an ultra-high-performance liquid chromatography (UHPLC) system (Vanquish, Thermo Fisher Scientific) coupled to a Q Exactive HFX mass spectrometer (Orbitrap MS, Thermo). Flow phase solution A: acetonitrile/water (60:40, v/v); flow phase solution B: acetonitrile/water (90:10, v/v), two flow phase solutions contain 10 mmol/L ammonium formate and 0.1% methanoic acid at final concentration. Then, a series of gradient solution B and solution A were eluted as follows: 95% solution B for 0.5 min, 70% solution B for 5 min, 50% solution B for 8 min, 40% solution B for 9 min, 70% solution A for 9 min and 95% solution A for 12 min. A mass spectrometer (Q Exactive HFX) was used to acquire MS/MS spectra data under the control of the acquisition software (Xcalibur, version 4.1, Thermo). Full scan MS spectra were continuously analyzed using the software. The parameters of electrospray used as the ionization (ESI) source conditions were as follows: sheath gas flow rate of 50 Arb, Aux gas flow rate of 10 Arb, capillary temperature of 320°C, full MS resolution of 60,000, MS/MS resolution of 7,500, collision energy of 10/30/60 in NCE mode, and spray voltage of 3.5 kV (positive model, ESI+) or −3.2 kV (negative model, ESI-) (22).

### Validation of candidate metabolites

To validate the applicability of the candidate metabolites, the ultrahigh-performance liquid chromatography–tandem mass spectrometer (UHPLC-MS/MS) was employed to quantitatively measure the candidate metabolites in the plasma of another 20 cases (10 cases in MT and MTs groups, respectively). The chromatographic separation was accomplished on an Agilent 1,290 Infinity II series UHPLC System (Agilent Technologies, California, USA), equipped with a Waters ACQUITY UPLC BEH Amide column (100 × 2.1 mm, 1.7 μm, Waters, USA). The mobile phase A was 1% formic acid with 20 mM ammonium formate in water, and phase B was 1% formic acid with 20 mM ammonium formate in acetonitrile. The column temperature and autosampler temperature were maintained at 35 and 4°C, respectively. The multiple reaction monitoring parameters of the target analytes are controlled by flowing injection of the standard solution of a single analyte.

### Data preprocessing and annotation

**Figure 1:**
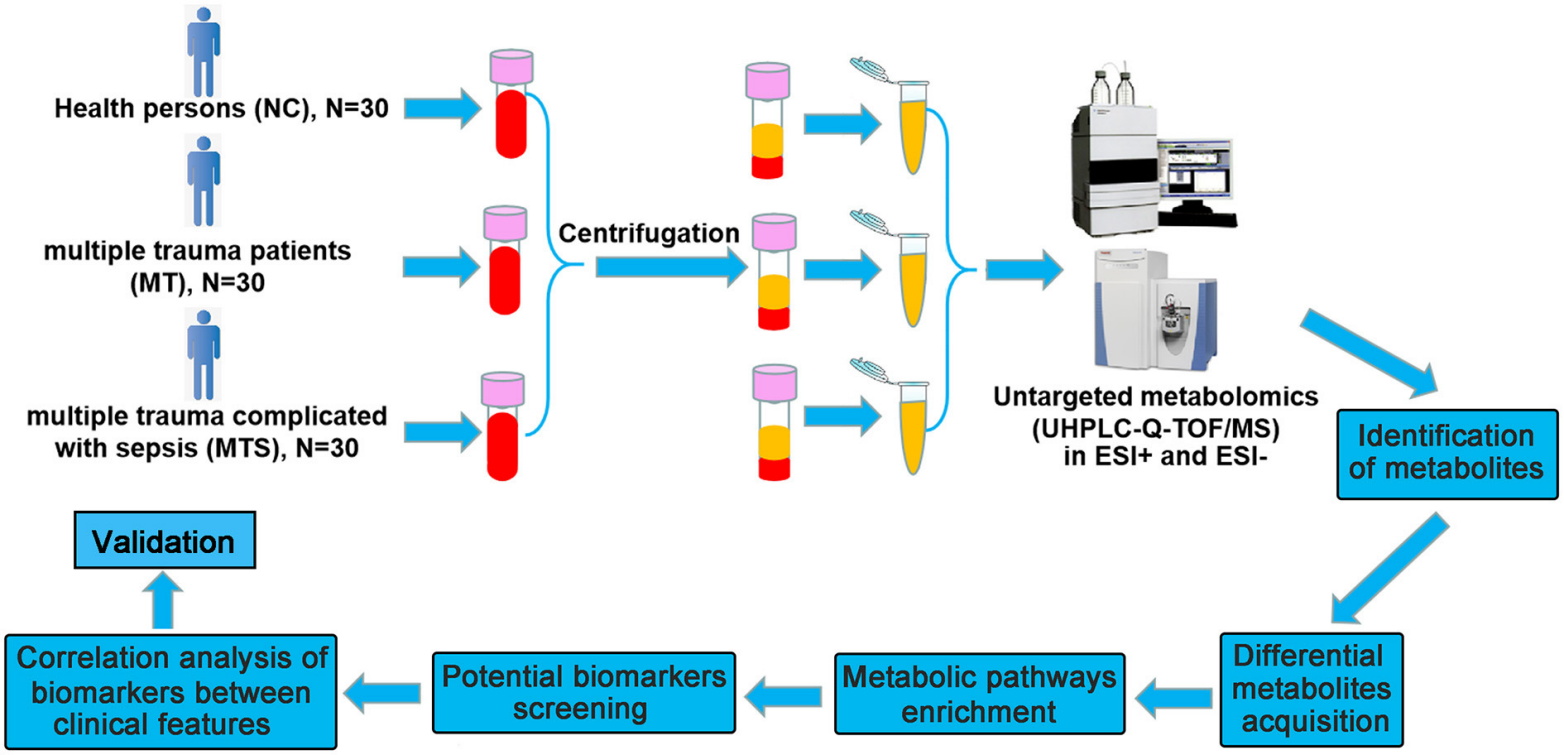
Schematic workflow for the experimental approach using untargeted metabolomics. The plasma were collected from NC, MT and MTS groups for metabolomics profiling using untargeted metabolomics. Quantitative information is extracted from MS data and identification based on database, and the metabolic pathways of differential metabolites were enriched and the potential biomarkers were further obtained, subsequently, the availability was predicted and the correlations between potential biomarkers and clinical characteristics were analyzed.

The raw data of peak were converted to mzXML format and detected by R package based on XCMS (version 3.2). A data matrix consisting of retention time (RT), Mass-to-charge ratio (m/z) values, and peak intensity was established by preprocessing. After discarding the data of QC samples, monoisotopic peaks were subjected to subsequent statistical analyses. Metabolites were identified and annotated using HMDB, METLIN, and MoNA databases, developed by Biotree Technology Co. Ltd. (Shanghai, China) (23). A schematic workflow of the study is shown in Figure 1.

### Statistical analysis

All statistical analyses were carried out using MetaboAnalyst 2.0 (http://www.metaboanalyst.ca). Principal component analysis (PCA, 95% confidence interval) was performed to visualize the distribution of sample groups and unsupervised multivariate statistical analysis. Orthogonal projections to latent structures-discriminate analysis (OPLS-DA) were performed as a supervised method to visualize group separation and identify significantly changed metabolites. In cross-validation and permutation tests, the OPLS-DA models were used according to multiple correlation coefficients (R2) and cross-validated R2 (Q2) value by 7-fold cross validation and 200 permutations. The principal component was obtained based on the importance of the projection (VIP) value determined using OPLS-DA analysis. Metabolites with VIP>1 and *P* < 0.05 (ANOVA) were considered significant differential metabolites among the groups. Pathway enrichment analysis was performed using KEGG (http://www.genome.jp/kegg/) and HMDB (http://www.hmdb.ca) databases (24, 25). Correlation analysis of metabolites and receiver operating characteristic (ROC) curves were drawn using GraphPad Prism 6.0. *P* < 0.05 was considered statistically significant.

## Results

### Patient demographics and clinical characteristics

This study enrolled 30 multiple trauma patients (MT) and 30 multiple trauma with sepsis (MTS) patients. The clinical characteristics of the patients are shown in Table 1. Forty-five patients (75%), including 22 MT patients and 23 MTS patients, were males. This showed that gender in MT and MTS groups has no difference (*P* = 1.0000). The median ages of the MT and MTS groups were 35 years (25th to 75th percentile, 26 to 46 years) and 44.5 years (25th to 75th percentile, 35.75 to 59.75 years), respectively. This showed the patient's age in MT and MTS groups has a significant difference (*P* = 0.0071). Although the MTS patients stayed in the ICU for more days than the MT patients, the median and *P* values were not different between the two groups. The MTS patients were more severe and had a significantly higher SOFA score (MTS: median; 2 points and 25th to 75th percentile; 1–6 points) than the MT group (MT: median; 0 points and 25th−75th percentile; 0–2 points) (*P* = 0.0061). The MTS patients also had a significantly lower Glasgow score (MTS: median; 12 points and 25th−75th percentile; 7.75–15 points) than the MT patients (MT: median; 15 points and 25th−75th percentile; 15–15 points) (*P* = 0.0007). The results indicated that the SOFA score and the Glasgow score could be quickly distinguish patients with MT and MTS in clinical. Furthermore, the median length of hospital stay [21 (15–17, 19, 21–40) *vs*. 23.5 (10.75–38.75), *P* = 0.1150], leukocyte count [15.47 (11.99–21.95) *vs*. 14.74 (11.14–18.02), *P* = 0.4362], neutrophil count [13.41 (9.497–19.42) *vs*. 12.91 (9.675–14.19), *P* = 0.2077], platelets [189.5 (154.5–256.3) *vs*. 183 (142.5–257), *P* = 0.4280], total bilirubin [10.55 (8.65–24.83) *vs*. 15.5 (9.45–22.95), *P* = 0.7753], creatinine [62.5 (52.78–68.23) *vs*. 64.9 (56.2–79.05), *P* = 0.2844], and oxygenation index [257 (181.3–389.6) *vs*. 271.2 (219.7–385.7), *P* = 0.3334] were not significantly different between the MTS and MT groups. Therefore, these results suggested that the age, SOFA score and Glasgow score may be related to the incidence of MTS.

**Table 1:** The basic information and clinical characteristics of multiple trauma with sepsis and multiple trauma without sepsis.

**Characteristics**	**Variables**			
	**All patients** **(*n* = 60)**	**Multiple trauma** **(MT, *n* = 30)**	**Multiple trauma with sepsis** **(MTS, *n* = 30)**	**P value** **(MT *vs*. MTS)**
Male gender, *n* (%)	45 (75%)	22 (73%)	23 (77%)	1.0000
Age, years	40 (31 to 52)	35 (26 to 46)	44.5 (35.75 to 59.75)	0.0071**
Length of stay in the ICU, days	0 (0 to 1)	0 (0 to 0)	0 (0 to 4.25)	0.5729
SOFA score, points	0 (0 to 2)	0 (0 to 2)	2 (1 to 6)	0.0061**
Length of stay in hospital, days	16 (9.5 to 29.5)	23.5 (10.75 to 38.75)	21 (11 to 34)	0.1150
Glasgow score, points	15 (11 to 15)	15 (15 to 15)	12 (7.75 to 15)	0.0007**
Leukocyte count, ×10^9^/L	14.74 (12 to 20.85)	14.74 (11.14 to 18.02)	15.47 (11.99 to 21.95)	0.4362
Neutrophil count, ×10^9^/L	12.91 (9.58 to 17.95)	12.91 (9.675 to 14.19)	13.41 (9.497 to 19.42)	0.2077
Platelets, ×10^9^/L	183 (149 to 256)	183 (142.5 to 257)	189.5 (154.5 to 256.3)	0.4280
Total bilirubin, μmol/L	12.7 (9.1 to 23.3)	15.5 (9.45 to 22.95)	10.55 (8.65 to 24.83)	0.7753
Creatinine, mg/dL	62.6 (54.6 to 75.9)	64.9 (56.2 to 79.05)	62.5 (52.78 to 68.23)	0.2844
Oxygenation index, mmHg	268.6 (209.7 to 385)	271.2 (219.7 to 385.7)	257 (181.3 to 389.6)	0.3334

Data are expressed as medians and 25th to 75th percentiles or with frequencies and percentages. P value is statistically significant when <0.01 and those values are marked with an ^**^.

ICU, intensive care unit; SOFA, sequential organ failure assessment.

### Assessment of metabolic profiles

**Figure 2:**
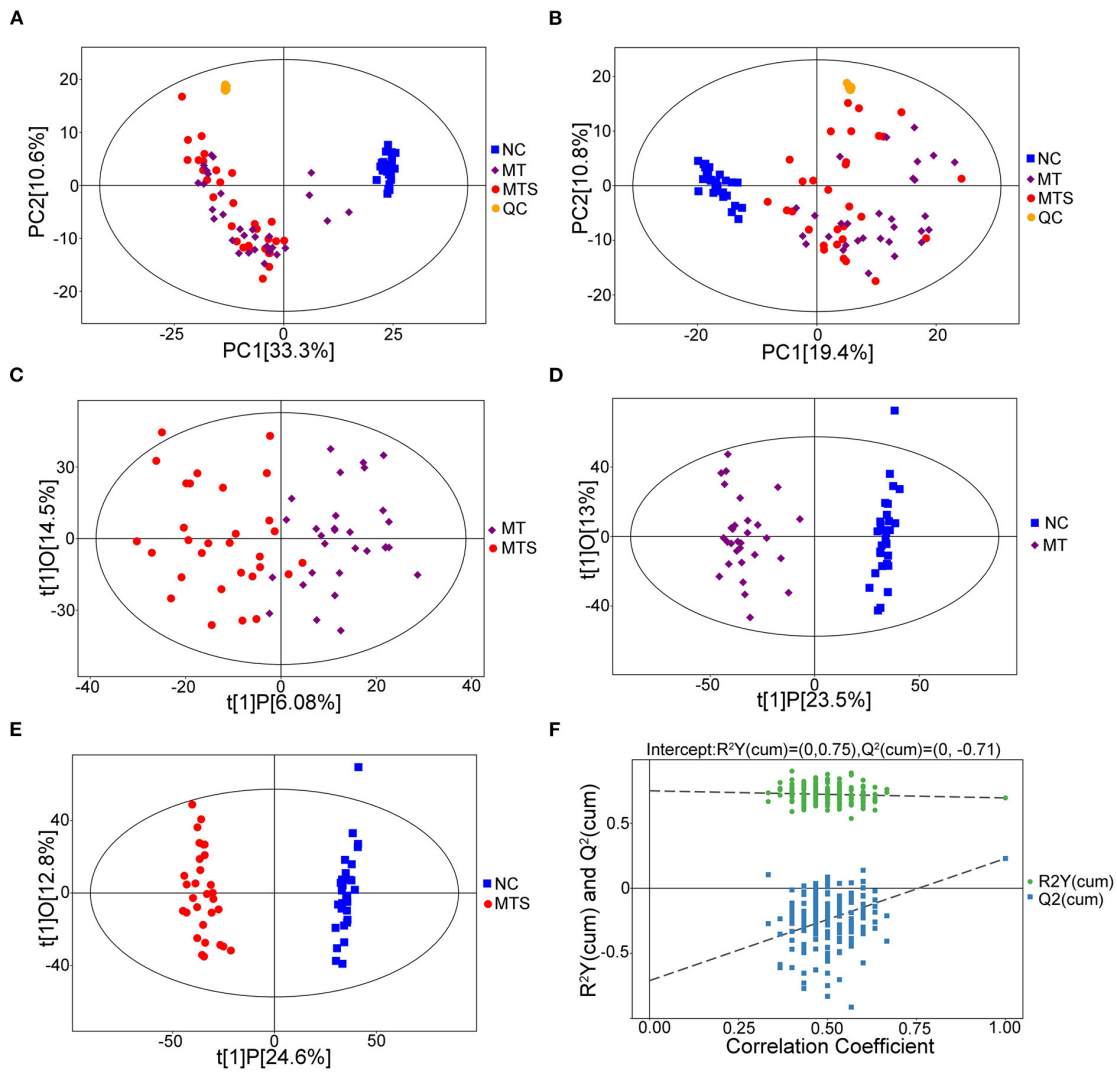
Metabolic profiles of plasma samples of NC, MT and MTS. **(A)** PCA score plots of the samples derived from the metabolite profiles in the ESI+ model, QC: quality control. **(B)** PCA score plots of the samples derived from the metabolite profiles in the ESI- model. **(C)** OPLS-DA score scatter plots of plasma samples of MTS *vs*. MT derived from the metabolite profiles in the ESI+ model. **(D)** OPLS-DA score scatter plots of plasma samples of MT *vs*. NC derived from the metabolite profiles in the ESI+ model. **(E)** OPLS-DA score scatter plots of plasma samples of MTS *vs*. NC derived from the metabolite profiles in the ESI+ model. **(F)** Permutation test of the OPLS-DA model for MTS *vs*. MT in the ESI+ model. *N* = 30 in each group. **(A–F)** were drawn by R version 4.0.2.

This study used untargeted metabolomics to assess the relationship between plasma metabolome and MTS. The UHPLC/MS profile of plasma samples for MTS, MT and NC in positive (ESI+) and negative (ESI-) modes are shown in Figure 2. A total of 5,168 peaks and 1,434 metabolites were identified and quantified in the ESI+ model, while 4,078 peaks and 847 metabolites were identified and quantified in the ESI- model. These compounds were annotated based on internal libraries and reference standards. The PCA score plot showed the NC, MT and MTS groups had different metabolic profiles. The ESI+ and ESI- models are shown in Figures 2A,B, respectively. The pairwise comparisons in the ESI+ and ESI– models is shown in Supplementary Figure 1. The orthogonal projections to latent structures discriminant analysis (OPLS-DA) were used to further assess the tendency of metabolite classification among the three groups. The OPLS-DA score plots of the MT *vs*. MTS groups (Figure 2C), NC *vs*. MT groups (Figure 2D), and NC *vs*. MTS groups (Figure 2E) in the ESI+ model suggested that the metabolites were reliable based on the differences between the groups. The OPLS-DA score plots of the MT *vs*. MTS groups (Supplementary Figure 2A), NC *vs*. MT groups (Supplementary Figure 2B), and NC *vs*. MTS groups (Supplementary Figure 2C) in the ESI- model also showed that the metabolites were reliable based on differences between the groups. Additionally, a random permutations test comparison between MT and MTS groups (ESI+) was performed to verify the validity and robustness of the OPLS-DA model. The negative corresponding Q2 value was used for the validation of the metabolic profiles (Figure 2F). Similarly, the comparison between the MT and MTS groups in the ESI- was valid (Supplementary Figure 2D).

### Differential metabolites obtained from the plasma of MTS patients

**Figure 3:**
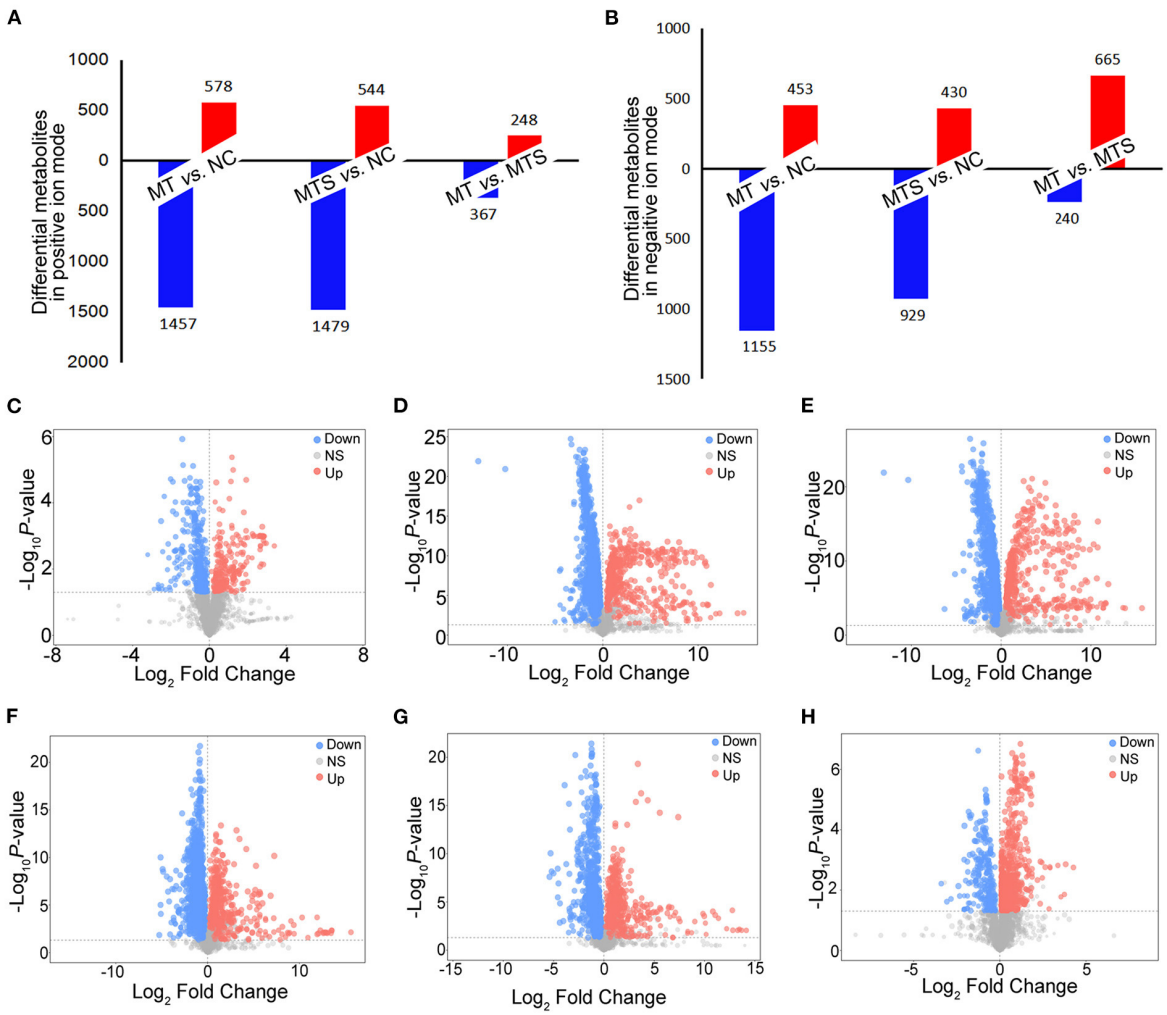
The distribution of differential plasma metabolites among NC, MT and MTS. **(A)** The number of differential plasma metabolites among NC, MT and MTS in the ESI+ model. **(B)** The number of differential plasma metabolites among NC, MT and MTS in the ESI- model. **(C)** Volcano plot of the MTS *vs*. MT groups in ESI+ model. **(D)** Volcano plot of the MT *vs*. NC groups in ESI+ model. **(E)** Volcano plot of the MTS *vs*. NC groups in the ESI+ model. **(F)** Volcano plot of the MTS *vs*. MT groups in ESI- model. **(G)** Volcano plot of the MT *vs*. NC groups in the ESI- model. **(H)** Volcano plot of the MTS *vs*. NC groups in the ESI- model. Each point in the volcano plot represents a significantly different metabolite, red represents upregulated metabolites, blue represents downregulated metabolites, and gray dots indicate non significant differences. **(C–H)** were drawn by R version 4.0.2.

This study used a pairwise comparison to screen the differential metabolites. The significantly differential metabolites were identified based on the criteria of variable importance of the projection (VIP) values >1.0 and *P* values < 0.05. A total of 1,457 metabolites were downregulated, and 578 were upregulated in the MT *vs*. NC, 1,479 metabolites were downregulated, and 544 were upregulated in the MTS *vs*. NC group, and 367 metabolites were downregulated, and 248 were upregulated in the MTS *vs*. NC group in the ESI+ model (Figure 3A). The volcano plots are shown in Figures 3C–E. A total of 1,155 metabolites were downregulated, and 453 were upregulated in the MT *vs*. NC group, 929 metabolites were downregulated, and 430 were upregulated in the MTS *vs*. NC group, and 240 metabolites were downregulated and 665 were upregulated in the MTS *vs*. NC group in the ESI- model (Figure 3B). The volcano plots are shown in Figures 3F–H.

### Detection and identification of differential metabolites

**Figure 4:**
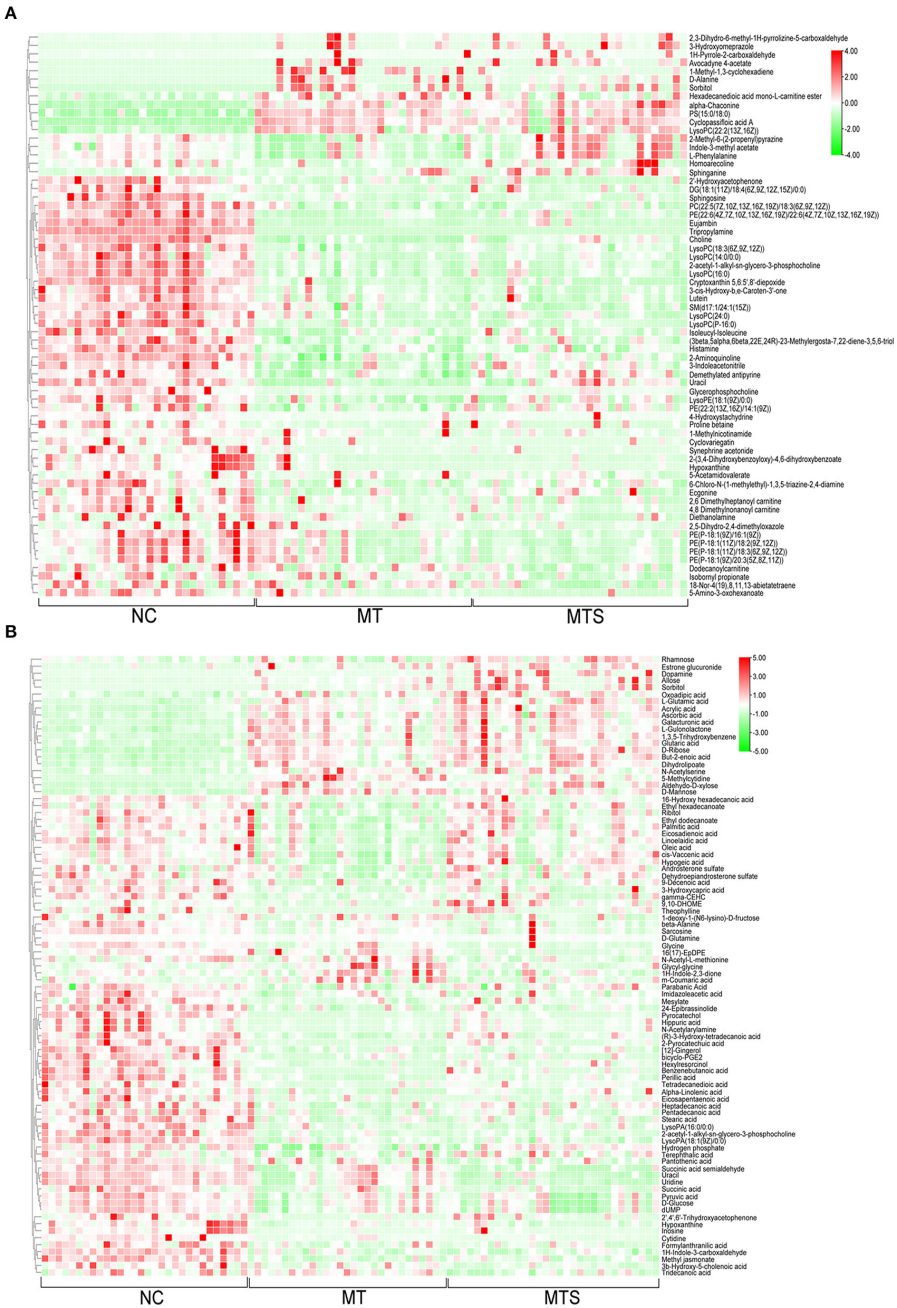
The hierarchical clustering heat map of metabolites from plasma of NC, MT and MTS groups in the ESI+ mode **(A)** and in the ESI- mode **(B)**. **(A,B)** were drawn by R version 4.0.2.

One-way ANOVA was used to compare all data in NC, MT and MTS groups based on the criteria of VIP values >1.0. The critical *P* value was set to 0.05 for significantly differential metabolites. A total of 156 significant plasma metabolites (67 in the ESI+ model and 89 in the ESI- model) were obtained (Supplementary Table 1). This study also conducted tentative identification of these metabolites and their corresponding concentration fold change analyses. Positive and negative fold changes represented upregulation and downregulation within comparative groups, respectively. A greater fold change of metabolites between pairwise comparisons and metabolites may be a better biomarker. The profiles of hierarchical clustering analysis were then visualized to assess the global overview of all the significantly differential metabolites in the ESI+ (Figure 4A) and ESI- models (Figure 4B).

### Pathway analysis of differential metabolites

**Figure 5:**
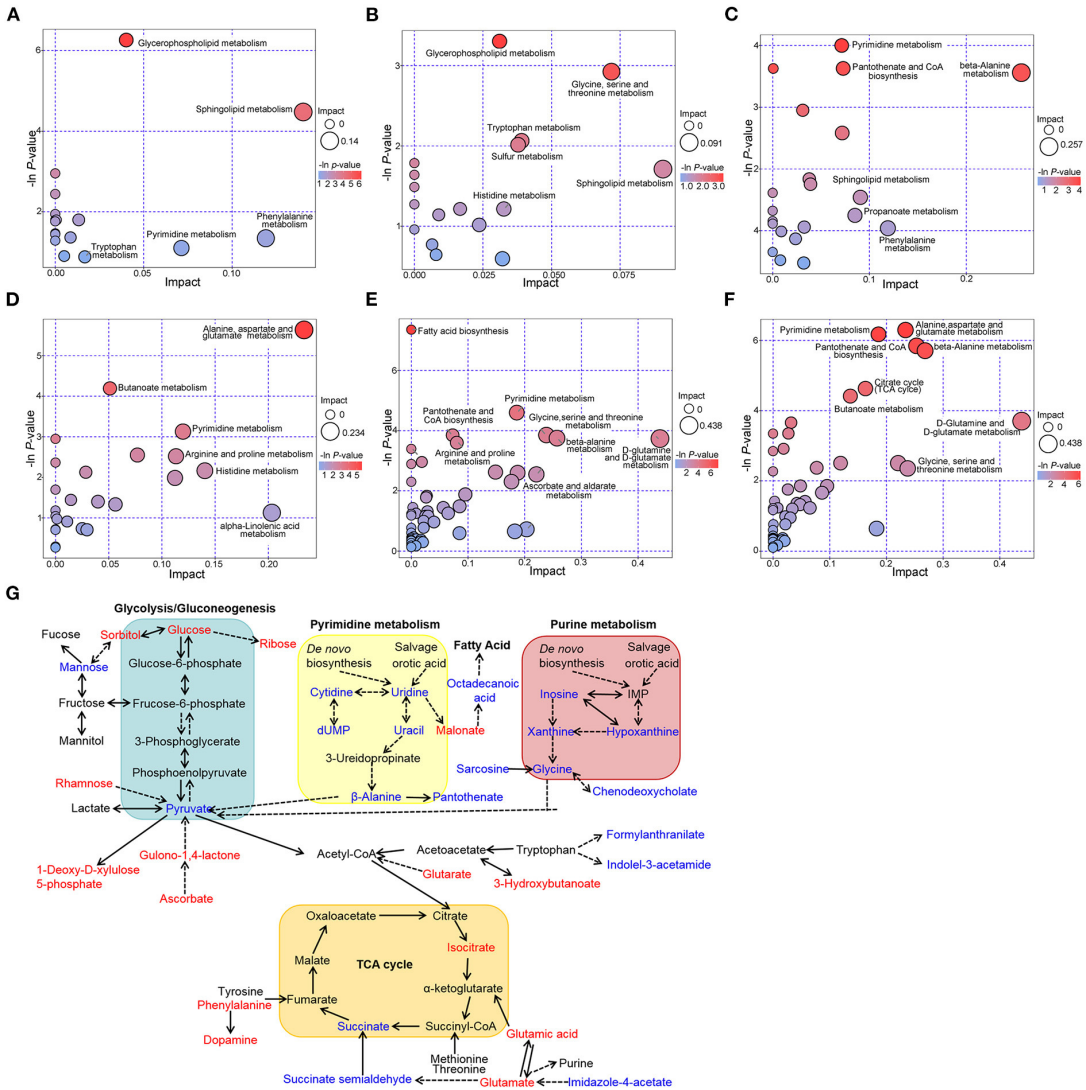
Metabolic pathways among NC, MT and MTS groups. Bubble diagram of the metabolic pathways of MTS *vs*. MT **(A)**, MT *vs*. NC **(B)**, and MTS *vs*. NC **(C)** in the ESI+ model. Bubble diagram of the metabolic pathways of MTS *vs*. MT **(D)**, MT *vs*. NC **(E)** and MTS *vs*. NC **(F)** in the ESI- model. The –ln(p) values from the pathway enrichment analysis are indicated on the horizontal axis, and impact values are indicated on the vertical axis. The colors and sizes of the shapes represent the effects of the pairwise comparison, and the larger red shapes indicate a greater effect on the pathway. **(G)** Schematic overview of the metabolites with plasma levels significantly altered in multiple trauma complicated with sepsis. Metabolites with increased levels are in red and those with decreased levels are in blue; Solid lines denote direct reactions; dotted lines denote indirect reactions; arrowhead indicates direction of the reaction; double arrowhead indicates direction of the reversible reactions. **(A–F)** were drawn by R version 4.0.2.

The enrichment analysis was conducted using the Kyoto Encyclopedia of Genes and Genomes (KEGG) pathway database to investigate the metabolites related to the metabolic pathways and physiological changes in the plasma of MTS patients. In the ESI+ model, glycerophospholipid, sphingolipid, tryptophan, pyrimidine, and phenylalanine metabolism pathways were affected in the MT *vs*. MTS group (Figure 5A); glycerophospholipid, glycine, serine, threonine, tryptophan, sulfur, sphingolipid, and histidine metabolism pathways were affected in the MT *vs*. NC group (Figure 5B); and pyrimidine, pantothenate and CoA biosynthesis, beta-alanine, sphingolipid, propanoate, and phenylalanine metabolism pathways were affected in the MTS *vs*. NC group (Figure 5C). In the ESI- model, alanine, aspartate, glutamate, butanoate, pyrimidine, arginine, proline, histidine, and alpha-linolenic acid metabolism pathways were affected in the MT *vs*. MTS group (Figure 5D); fatty acid biosynthesis, glycine, serine, threonine, pyrimidine, pantothenate and CoA biosynthesis, beta-alanine, arginine, proline, ascorbate, aldarate, D-glutamine and D-glutamate metabolism pathways were affected in the MT *vs*. NC group (Figure 5E); and pyrimidine, alanine, aspartate, glutamate, pantothenate and CoA biosynthesis, beta-alanine, citrate cycle (TCA cycle), butanoate, D-glutamine and D-glutamate, glycine, serine, and threonine metabolism pathways were affected in the MTS *vs*. NC group (Figure 5F). In general, these differentially altered metabolites were enriched in amino acid metabolism, lipid metabolism, glycometabolism, and nucleotide metabolism as shown in Figure 5G.

### Screening of potential biomarkers

**Figure 6:**
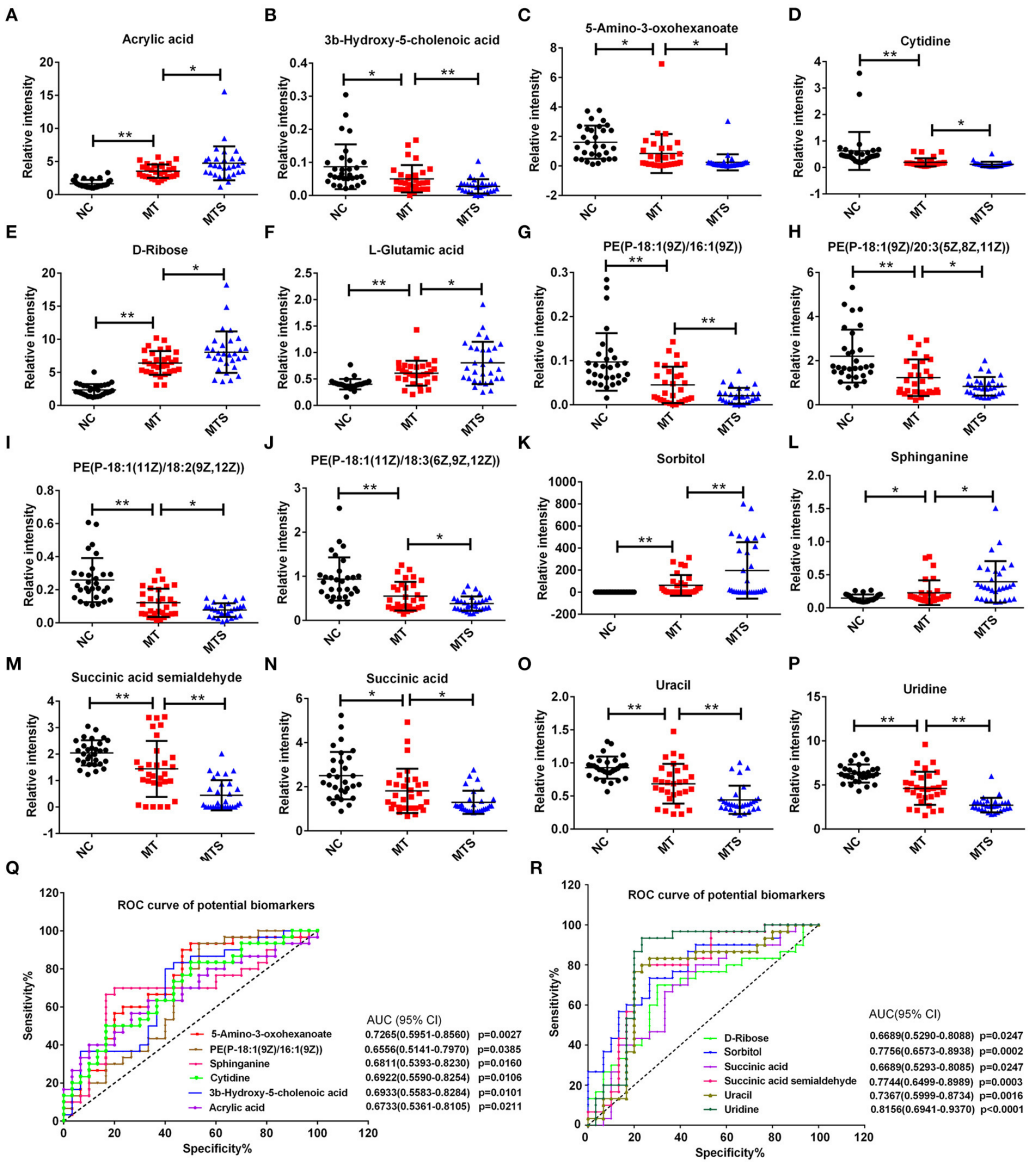
Scatter and trend plot of 16 potential biomarkers in the ESI+ and ESI- models. The scatter and trend plot of acrylic acid **(A)**, 3b-hydroxy-5-cholenoic acid **(B)**, 5-amino-3-oxohexanoate **(C)**, cytidine **(D)**, D-ribose **(E)**, L-glutamic acid **(F)**, PE [P-18:1(9Z)/16:1(9Z)] **(G)**, PE [P-18:1(9Z)/20:3(5Z,8Z,11Z)] **(H)**, PE [P-18:1(11Z)/18:2(9Z,12Z)] **(I)**, PE [P-18:1(11Z)/18:3(6Z,9Z,12Z)] **(J)**, sorbitol **(K)**, sphinganine **(L)**, succinic acid semialdehyde **(M)**, succinic acid **(N)**, uracil **(O)**, and uridine **(P)**, and the ordinate was the relative intensity of metabolite. **(Q,R)** Receiver operator curve (ROC) analysis of the random forest model combining 12 biomarkers (*P* < 0.05) to diagnose MTS in the validation data. *P < 0.05, **P < 0.01.

This study used 16 of the 156 differential metabolites to discriminate MT and MTS. The 16 metabolites were selected based on an increasing or decreasing trend from NC, MT to MTS, and significant differences in pairwise comparison to better distinguish the potential of MT patients to develop MTS (Figures 6A–P). Notably, acrylic acid, 3b-hydroxy-5-cholenoic acid, 5-amino-3-oxohexanoate, cytidine, D-ribose, L-glutamic acid, PE [P-18:1(9Z)/16:1(9Z)], PE [P-18:1(9Z)/20:3(5Z,8Z,11Z)], PE [P-18:1(11Z)/18:2(9Z,12Z)], PE [P-18:1(11Z)/18:3(6Z,9Z,12Z)], sorbitol, sphinganine, succinic acid semialdehyde, succinic acid, uracil, uridine, sphinganine, and succinic acid semialdehyde (MTS) had clear criteria for the progression of MT to MTS. Furthermore, receiver operating characteristic (ROC) curves were used to predict the class of subjects in the validation with a random forest (RF) model based on the data of the MT and MTS groups to evaluate the diagnostic potential of these metabolic biomarkers for MTS patients. The area under the curves (AUC) of 5-amino-3-oxohexanoate, PE [P-18:1(9Z)/16:1(9Z)], sphinganine, cytidine, 3b-hydroxy-5-cholenoic acid, acrylic acid, D-ribose, sorbitol, succinic acid, succinic acid semialdehyde, uracil, and uridine are shown in Figures 6Q,R. Notably, the RF model based on the 12 biomarkers with significant differences showed good diagnostic performance in MTS patients.

### Correlations between metabolites and clinical variables

This study used Spearman's correlation between the above 16 statistically significant metabolites and clinical variables (age, SOFA score, and Glasgow score) to determine the clinical availability of potential biomarkers further. The *P*-value and correlations (r) are shown in Table 2. The 5-amino-3-oxohexanoate, 3b-hydroxy-5-cholenoic acid, cytidine, succinic acid semialdehyde, uracil and uridine were negatively correlated with the SOFA score. In contrast, acrylic acid was positively correlated with the SOFA score. The 5-amino-3-oxohexanoate, PE [P-18:1(9Z)/16:1(9Z)], cytidine, 3b-hydroxy-5-cholenoic acid, succinic acid semialdehyde, uracil, and uridine were positively correlated with the Glasgow score, while sphinganine was negatively correlated with the Glasgow score. Moreover, uridine was negatively correlated with age. Therefore, the 9 noteworthy candidate biomarkers that are correlated with clinical variables may be suitable for the clinical diagnosis of MTS.

**Table 2:** Correlations between metabolites and clinical variables.

**Metabolite**	**Clinical Variable**					
	**Age**		**SOFA score**		**Glasgow score**	
	***r***	***P* value**	***r***	***P* value**	***r***	***P* value**
5-Amino-3-oxohexanoate	0.03742	0.7882	−0.2903	0.0315*	0.3252	0.0154*
PE [P-18:1(11Z)/18:2(9Z,12Z)]	−0.1111	0.4237	−0.07689	0.5769	0.2093	0.1251
PE [P-18:1(11Z)/18:3(6Z,9Z,12Z)]	−0.1174	0.398	−0.1494	0.2763	0.198	0.1473
PE [P-18:1(9Z)/16:1(9Z)]	−0.04119	0.7674	−0.1109	0.4203	0.337	0.0119*
PE [P-18:1(9Z)/20:3(5Z,8Z,11Z)]	−0.1571	0.2567	−0.1816	0.1844	0.185	0.1764
Sphinganine	0.08383	0.5467	0.2177	0.1103	−0.3101	0.0212*
3b-Hydroxy-5-cholenoic acid	−0.04752	0.7329	−0.3202	0.0172*	0.329	0.0142*
Acrylic acid	0.07945	0.568	0.2844	0.0353*	−0.09063	0.5105
Cytidine	−0.1624	0.2408	−0.3447	0.01*	0.2979	0.0272*
D-Ribose	0.1142	0.411	0.2382	0.0799	−0.1915	0.1612
L-Glutamic acid	0.22	0.1099	0.1458	0.2881	−0.1835	0.1799
Sorbitol	0.2203	0.1094	−0.1351	0.3255	0.01953	0.8875
Succinic acid	−0.2013	0.1444	−0.2231	0.1015	0.2217	0.1038
Succinic acid semialdehyde	−0.2483	0.0702	−0.3452	0.0099**	0.3682	0.0057**
Uracil	−0.08372	0.5473	−0.3425	0.0105*	0.3881	0.0034**
Uridine	−0.3173	0.0194*	−0.4536	0.0005**	0.396	0.0028**

The P < 0.05 were considered significant and marked with ^*^, and P < 0.01 were marked with ^**^. SOFA, sequential organ failure assessment.

### Identification of MTS biomarkers by UPHLC-MS/MS targeted quantitative analysis

**Figure 7:**
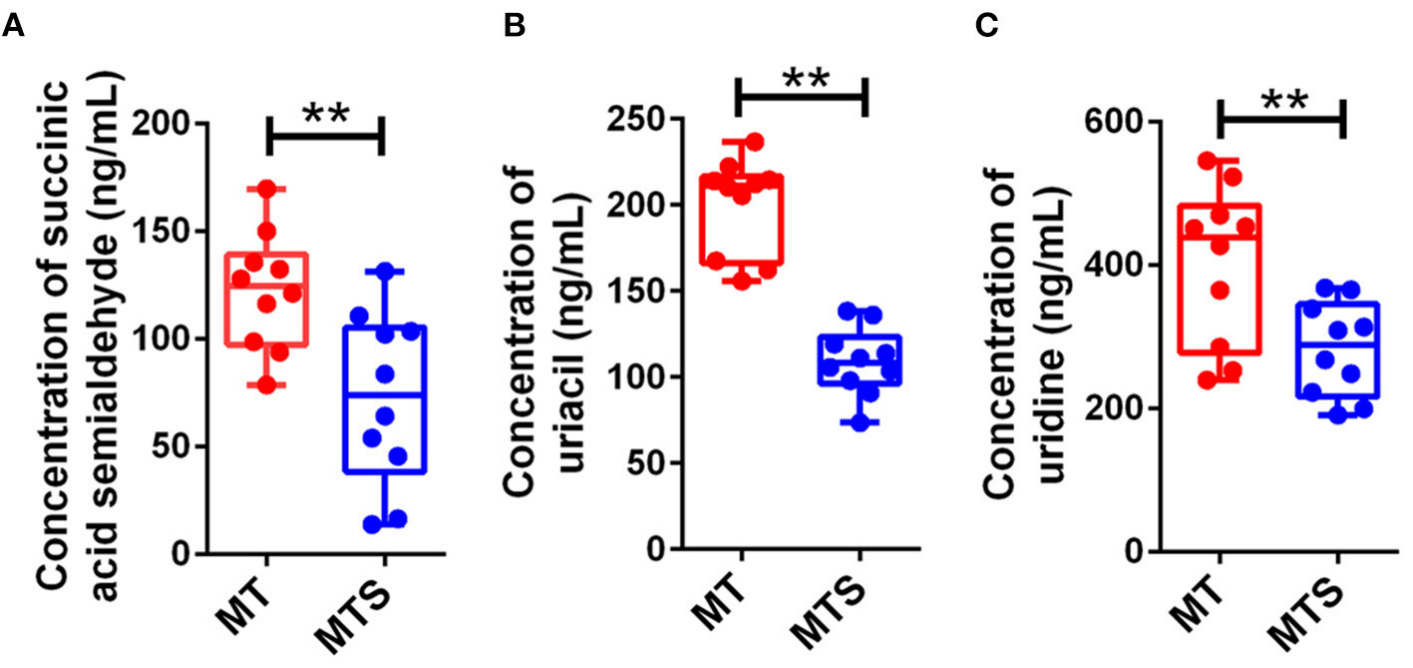
Candidate biomarkers were identified by UHPLC-MS/MS quantitative analysis in another batch of 20 cases (*n* = 10 in MT and MTS groups, respectively). **(A)** Succinic acid semialdehyde. **(B)** Uracil. **(C)** Uridine. **P < 0.01.

To validate the 9 candidate metabolites (acrylic acid, 5-amino-3-oxohexanoate, 3b-hydroxy-5-cholenoic acid, cytidine, succinic acid semialdehyde, PE [P-18:1(9Z)/16:1(9Z)], sphinganine, uracil, and uridine) could accurately distinguish MTS from MT, another batch of 20 cases contains MT and MTS groups (10 cases in each group) was examined by UHPLC-MS/MS quantitative analysis. The results showed that succinic acid semialdehyde, uracil, and uridine had significant differences (Figure 7). Therefore, these results suggest that the three metabolites could be used as potential diagnostic biomarkers in MTS patients.

## Discussion

Multiple trauma complicated with sepsis is one of the causes of high mortality in the ICU. Therefore, timely monitoring of sepsis progression in posttraumatic patients is crucial in MT treatment (26). Studies have shown that MT is the major risk factor for sepsis development. Moreover, early sepsis diagnosis can prevent septic progression. However, the physiological mechanisms of sepsis are unknown. Furthermore, it is difficult to identify early biomarkers of sepsis. This study aimed to identify biomarkers of sepsis for early diagnosis using metabolomics analysis techniques. Metabolomics is a promising area of research because metabolome changes are more dynamic than the genome, and proteome changes quickly. Besides, metabolite changes can directly reflect the changes in many small molecules, such as nucleotides, amino acids, and lipids (27, 41). Although various studies have used metabolomics to screen biomarkers of trauma complicated with sepsis, these metabolites can only be used as diagnostic indicators and not for early diagnosis since these potential biomarkers are compared with normal people and MT patients (28, 42). Moreover, the identified diagnostic biomarkers were not specific for MTS. Although there are some advances in the metabolomics of sepsis, some factors still limit the clinical application of metabolomics. These biomarker candidates have failed validation in confirmation studies. Therefore, besides healthy controls, the design strategy of biomarker screening should also include controls with non-related diseases (29, 30, 32, 33). This study used plasma samples of healthy persons (NC), multiple trauma (MT), and multiple traumas complicated with sepsis (MTS) patients for metabolomics analysis. This study used UHPLC-MS for metabolomics detection. Previously, one-dimensional (1-D) proton (H) nuclear magnetic resonance (1H-NMR) was applied in metabolomics of sepsis, a recent study used 1H-NMR-based metabolomics to analyze and screen potential biomarkers for early diagnosis of metabolite concentrations between serum septic patients and healthy controls. The study showed that glucose, glycine, 3-hydroxybutyrate, creatinine and glycoprotein acetyl levels are higher in sepsis patients than in healthy controls. In contrast, citrate and histidine levels are lower in sepsis patients than in healthy controls (28). Although nuclear magnetic resonance (1H-NMR) and mass spectrometry (MS) combined with multivariate analysis can be used for sepsis metabolomics analysis, but MS has a greater sensitivity than NMR and presented a wider application prospect (31). Additionally, MS can accurately determine and quantify molecules and provide structural information of the detected compounds (43). Therefore, this study obtained several differential metabolites using UHPLC-MS technology, verifying its sensitivity and practicability.

Male sex, SOFA score and Glasgow score were the observably independent risk factors for the development of posttraumatic sepsis. A similar study showed that the age of patients and days of stay in the ICU are significantly different between sepsis (*n* = 9) and no sepsis (*n* = 12) groups (32). Furthermore, a study assessed 29,829 patients in Germany and showed that various factors, including male sex, preexisting medical condition, Glasgow Coma Scale score, Injury Severity Score, number of transfused red blood cell units, and number of operative procedures, are independent risk factors for the traumatic sepsis development. Additionally, the MTS patients have a longer stay in ICU, higher rates of organ failure and hospital mortality than the non-sepsis patients (33). Analogously, a systematic review involving 56,164 patients found that demographic factors, such as old age and male sex, are associated with an increased risk of sepsis (44). Herein, only age, SOFA score and Glasgow score were significantly different between the two groups, possibly due to the small sample size. Studies have reported that the incidence of sepsis is increased in elderly adults and age is an independent predictor of sepsis mortality (34). SOFA score has been widely used in septic evaluation, showing a moderate prognostic stratification ability (45). The Glasgow coma scale has been incorporated into the new sepsis recommendation (Sepsis 3.0). It can also be used to evaluate the mental state of patients with sepsis since sepsis can induce central nervous system infection and diffuse brain dysfunction (46). Therefore, these studies support our results of clinical characteristics to some extent.

Sepsis researches focus on exploring an ideal biomarker. Researchers have been exploring an ideal biomarker that can quickly and sensitively distinguish the presence and progression of sepsis. The early clinical diagnosis of sepsis depends on the presence of microbiologic cultures in blood. However, positive results are detected in only 30% of patients with sepsis or septic shock. As a result, studies have focused on the effects of metabolites produced by sepsis on individuals. Several biomarkers, such as procalcitonin, C-reactive protein, interleukin (IL)-6, and other inflammatory factors, have been proposed for sepsis detection (47). However, the clinical use of the existing biomarkers is limited. Although advances have been made in biomarkers for sepsis diagnosis, no single biomarker can meet the needs of specificity and sensitivity to distinguish sepsis from other inflammatory processes. Therefore, omics techniques, especially metabolomics, have been used to identify new biomarkers for sepsis progression. Although some studies use transcriptome or proteomics to screen sepsis biomarkers, metabolomics based on UHPLC-MS can also assess the effects of transcription and translation levels *in vivo*. Moreover, non-targeted metabolomics can systematically and comprehensively evaluate the unknown mechanism (35, 36). Therefore, metabolomics may play a critical role in the identification of sepsis biomarkers. Besides, the combined analysis of metabolomics and transcriptome or proteomics may be an important direction for the discovery of sepsis biomarkers in the future. Human serum, plasma and urine samples can be used to study the metabolome of sepsis to find promising biomarkers. Although various studies have used LC/MS techniques for sepsis metabolomics, the differential metabolites obtained in each study are different, possibly due to the resolution of the mass spectrometer used and the cause of sepsis.

Herein, 1,520 differential metabolites were detected between MT and MTS from the plasma of patients. The metabolites were enriched in amino acid metabolism, glycometabolism, lipid metabolism, nucleotide metabolism and other metabolic pathways. Some amino acids were lower in MTS patients than in the MT or NC groups, indicating that protein metabolism is a consumption process of amino acid in sepsis. The heterogeneity of the etiology of sepsis may lead to the different differential metabolites or potential biomarkers obtained *via* metabolomics in the early sepsis diagnosis. However, further studies are needed to assess the confirmation and regulatory mechanisms of these potential biomarkers in the clinical diagnosis of sepsis. The lower levels of glucose and organic acids, such as succinic acid, glutaric acid and pyruvic acid suggested that the citrate cycle (TCA cycle) and glycolysis/gluconeogenesis metabolism were disturbed in the MTS group. The intermediate products of the TCA cycle were significantly changed in the sepsis group than in the NC and MT groups, indicating that energy metabolism was disturbed in the sepsis group, thus decreasing energy production. An omic technologies review showed that sepsis affected the intermediate metabolite levels of the TCA cycle and is associated with mitochondrial beta-oxidation dysfunction of fatty acid metabolism (37). Furthermore, the inhibition of the TCA cycle requires energy supply through the anaerobic respiration pathway of glycolysis, leading to the conversion of pyruvate to alanine. The TCA cycle of mitochondria is the main pathway for the conversion of glutamine to CO_2_ and pyruvate (38). Mannose levels were higher in the plasma of the MTS and MT groups than the mannose levels in the NC group, and the alteration was enriched in mannose metabolism (15). Moreover, numerous lipids and lipid-like molecules were significantly affected in the MTS group compared with the MT or NC group. These differential metabolites were enriched in metabolic pathways of glycerophospholipid, sphingolipid, alpha-linolenic acid, arachidonic acid, linoleic acid, fatty acid, and fatty acid biosynthesis. A similar study showed that glycerophospholipids and sphingolipids are altered in sepsis patients (39). Lipids are involved in the initiation and regression of septic inflammation (40). Herein, unsaturated fatty acids, such as linoleic acid, alpha-linolenic acid and arachidonic acid, were significantly affected in the MTS group compared with the MT and NC groups. The double bonds of polyunsaturated fatty acids are attacked by oxidative stress in lipids. Moreover, peroxides and aldehydes generate chain reactions involved in lipid peroxidation-related signaling pathways associated with deleterious consequences (48). Molecules with anti-inflammatory properties have been found in omega-3 fatty acids eicosapentaenoic acid, docosahexaenoic acid, and arachidonic acid (49). A study also showed that oxidative stress and lipid metabolism promote sepsis development (50). Furthermore, other pathways, including urea cycle metabolism, glutathione metabolism, and primary bile acid biosynthesis, were also affected in the MTS group. These pathways play important roles in sepsis (51, 52). Therefore, this study provides evidence for the relationship between glucose or lipid metabolism and sepsis. Herein, the age of patients was significantly correlated with uridine, possibly due to the decreased expression of uridine phosphorylases in the aged, destroying uridine homeostasis (53). Glasgow coma scale was used to evaluate coma status in patients with sepsis. The results showed the Glasgow score was significantly correlated with some metabolites, including some central nervous system-related metabolites. Cytidine, uracil, uridine and sphingosine are associated with sepsis (15, 54–56). In this study, the 9 candidate metabolites was finally examined to quantitative analysis and the results suggested that succinic acid semialdehyde, uracil, and uridine could be used as potential diagnostic biomarkers in MTS patients. Although these published studies support the reliability of the metabonomic results, the usefulness and reprocibility of these novel biomarkers should be further confirmed depend on larger sample size in clinical.

In conclusion, this study identified three metabolic markers for MTS diagnosis after various analyses through untargeted plasma metabolomics. Meanwhile, these biomarkers may be used to screen MTS and assess the state of heterogeneous sepsis patients. However, this study has some limitations. (a) This study had a small sample size; However, the study randomly screened and enrolled eligible patients into the cohort to reduce errors. (b) This study did not confirm whether these metabolic variates are related to the early sepsis stage. (c) The biomarkers were not specific to all sepsis patients due to the genetic polymorphisms and host differences. Therefore, larger cohort study studies are needed to verify the results and improve the understanding of the pathophysiology of trauma-induced sepsis, providing a basis for managing traumatized patients in the ICU, including early diagnosis, targeted therapy and follow-up investigation.

## Data availability statement

The original contributions presented in the study are included in the article/Supplementary material, further inquiries can be directed to the corresponding author/s.

## Ethics statement

The studies involving human participants were reviewed and approved by Ethics Committee of the General Hospital of Ningxia Medical University. The patients/participants provided their written informed consent to participate in this study.

## Author contributions

KF, WD, LL, SL, YG, and ZC performed the experimental research and data analysis. XF wrote and edited the manuscript. GC and XF contributed to the study design, data analysis, and writing and editing of the manuscript. All authors read and approved the final manuscript and, therefore, had full access to all the data in the study and take responsibility for the integrity and security of the data.

## Funding

This work was supported by the Key Research and Development Program of Ningxia (2022BEG02049 and 2022BEG03084).

## Conflict of interest

The authors declare that the research was conducted in the absence of any commercial or financial relationships that could be construed as a potential conflict of interest.

## Publisher's note

All claims expressed in this article are solely those of the authors and do not necessarily represent those of their affiliated organizations, or those of the publisher, the editors and the reviewers. Any product that may be evaluated in this article, or claim that may be made by its manufacturer, is not guaranteed or endorsed by the publisher.
