# Supplementary material for: Identification of biomarkers and the mechanisms of multiple trauma complicated with sepsis using metabolomics
Source: Front Public Health. 2022 Aug 4;10:923170. doi: 10.3389/fpubh.2022.923170 (PMC9387941; doi:10.3389/fpubh.2022.923170)
Supplement: Supplementary file 1 [file Table_1.DOCX]

Supplementary Material


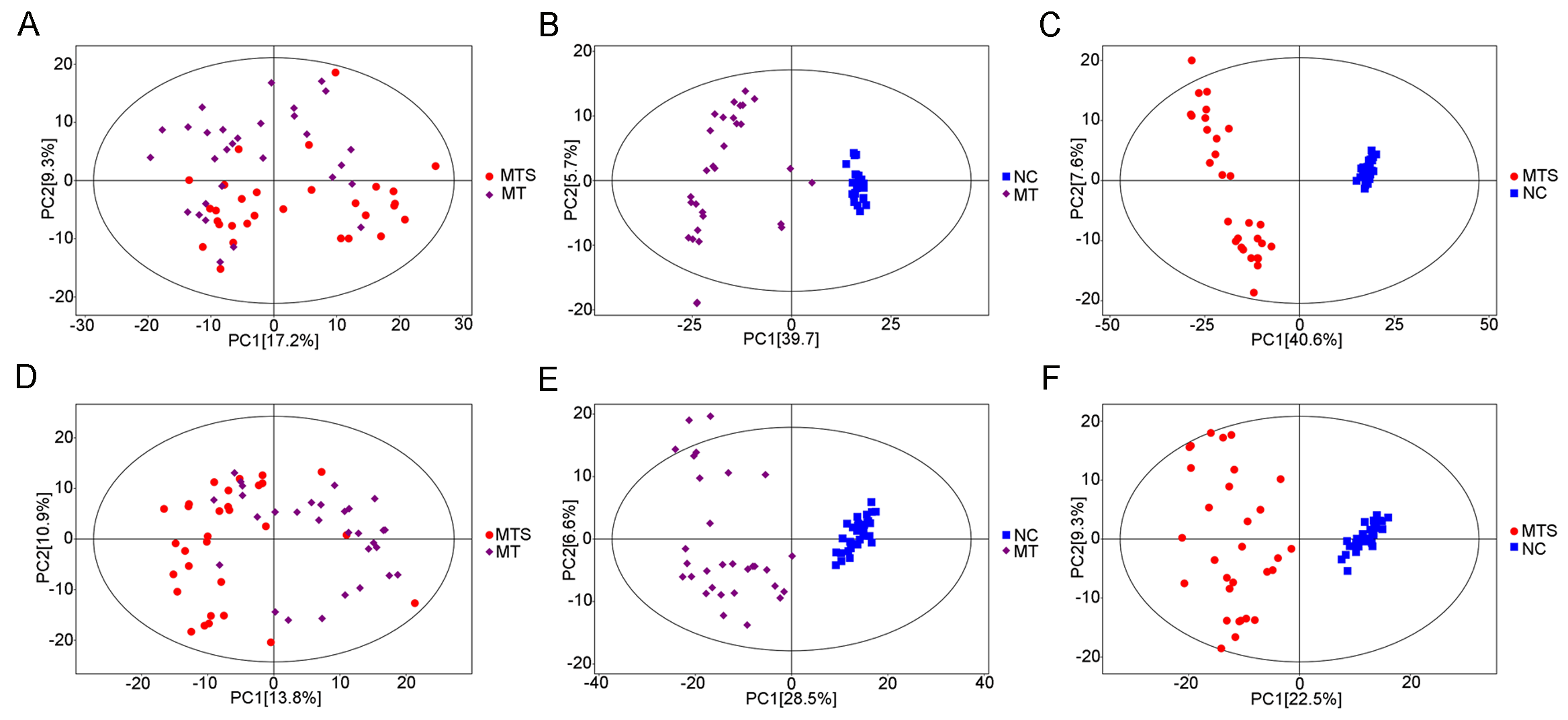


**Supplementary Figure 1**. The PCA score plots of the samples derived from the metabolite profiles. **(A)** The PCA score plots of MTS *vs*. MT in the ESI+ model. **(B)** The PCA score plots of MT *vs*. NC in the ESI+ model. **(C)** The PCA score plots of MTS *vs*. NC in the ESI+ model. **(D)** The PCA score plots of MTS *vs*. MT in the ESI- model. **(E)** The PCA score plots of MT *vs*. NC in the ESI- model. **(F)** The PCA score plots of MTS *vs*. NC in the ESI- model.


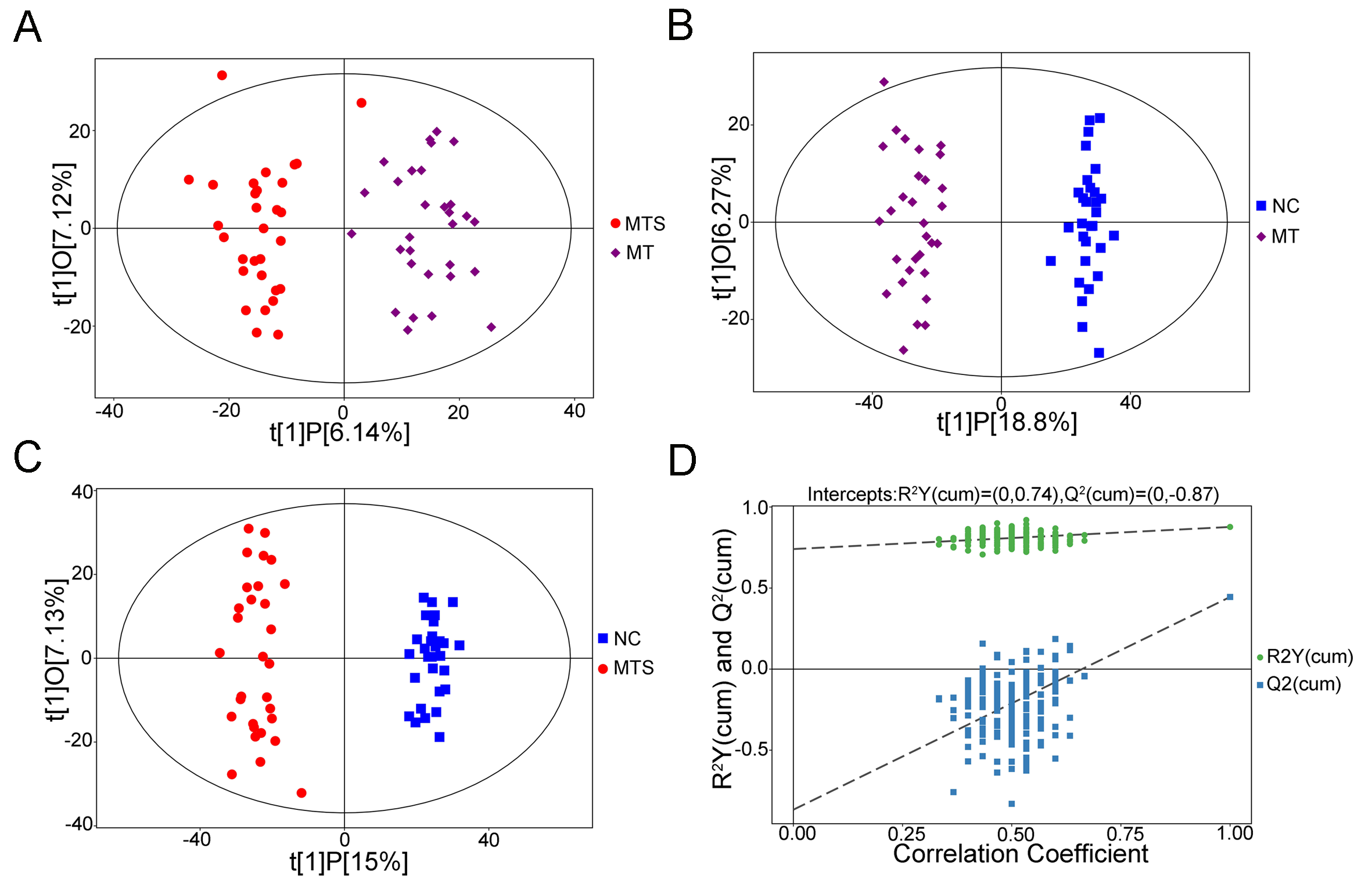


**Supplementary Figure 2**. The OPLS-DA score scatter plots and Permutation test among NC, MT and MTS in ESI- model. **(A)** OPLS-DA score scatter plots of plasma samples of MTS *vs*. MT derived from the metabolite profiles in ESI- model. **(B)** OPLS-DA score scatter plots of plasma samples of MT *vs*. NC derived from the metabolite profiles in ESI- model. **(C)** OPLS-DA score scatter plots of plasma samples of MTS *vs*. NC derived from the metabolite profiles in ESI- model. **(D)** Permutation test of the OPLS-DA model for the MTS *vs.* MT in ESI- model. N = 30 in each group.

| **Supplementary Table 1** Identification results of candidate biomarkers in plasma related to multiple trauma with sepsis. | | | | | | | | | | |
| --- | --- | --- | --- | --- | --- | --- | --- | --- | --- | --- |
| **Metabolite**  **NO.** | **Ionization mode** | **MS2 name** | **RT(s)** | **m/z** | **SuperClass** | **ANOVA P-Value** | **Q-Value** | **Log2 Fold Change** | | |
|  |  |  |  |  |  |  |  | **MTS *vs*. MT** | **MT *vs*. NC** | **MTS *vs*. NC** |
| 1 | ESI (+) | (3beta,5alpha,6beta,22E,24R)-23-Methylergosta-7,22-diene-3,5,6-triol | 31.1074 | 445.3671 | Lipids and lipid-like molecules | 0.0000 | 0.0000 | -0.4358 | -0.7918 | -1.2276 |
| 2 | ESI (+) | 18-Nor-4(19),8,11,13-abietatetraene | 27.6416 | 255.2104 | Lipids and lipid-like molecules | 0.0015 | 0.0027 | 0.8032 | -1.0290 | -0.2259 |
| 3 | ESI (+) | 1H-Pyrrole-2-carboxaldehyde | 89.602 | 96.0447 | Organic oxygen compounds | 0.0311 | 0.0444 | 0.3987 | 3.4090 | 3.8077 |
| 4 | ESI (+) | 1-Methyl-1,3-cyclohexadiene | 423.33 | 95.0859 | Hydrocarbons | 0.0001 | 0.0003 | 2.8047 | 5.0677 | 7.8725 |
| 5 | ESI (+) | 1-Methylnicotinamide | 336.444 | 137.0709 | Organoheterocyclic compounds | 0.0024 | 0.0042 | 0.9879 | -0.9549 | 0.0330 |
| 6 | ESI (+) | 2-(3,4-Dihydroxybenzoyloxy)-4,6-dihydroxybenzoate | 231.5495 | 307.0436 | Phenylpropanoids and polyketides | 0.0000 | 0.0000 | 1.2438 | -3.1914 | -1.9477 |
| 7 | ESI (+) | 2,3-Dihydro-6-methyl-1H-pyrrolizine-5-carboxaldehyde | 49.3613 | 150.0911 | Organoheterocyclic compounds | 0.0125 | 0.0193 | 0.0731 | 3.0523 | 3.1254 |
| 8 | ESI (+) | 2,5-Dihydro-2,4-dimethyloxazole | 53.7095 | 100.0759 | Organoheterocyclic compounds | 0.0056 | 0.0093 | 1.1772 | -1.9380 | -0.7608 |
| 9 | ESI (+) | 2,6 Dimethylheptanoyl carnitine | 221.079 | 302.2319 | Lipids and lipid-like molecules | 0.0003 | 0.0006 | 0.2614 | -1.2530 | -0.9915 |
| 10 | ESI (+) | 2-acetyl-1-alkyl-sn-glycero-3-phosphocholine | 215.0235 | 524.3696 | Lipids and lipid-like molecules | 0.0000 | 0.0000 | -0.1970 | -1.3437 | -1.5407 |
| 11 | ESI (+) | 2-Aminoquinoline | 60.6078 | 145.0759 | Organoheterocyclic compounds | 0.0000 | 0.0000 | -0.3410 | -0.6361 | -0.9770 |
| 12 | ESI (+) | 2'-Hydroxyacetophenone | 25.8105 | 137.0595 | Benzenoids | 0.0000 | 0.0000 | -0.0765 | -1.7524 | -1.8289 |
| 13 | ESI (+) | 2-Methyl-6-(2-propenyl)pyrazine | 234.174 | 135.0916 | Organoheterocyclic compounds | 0.0000 | 0.0001 | -0.6347 | 0.1747 | -0.4601 |
| 14 | ESI (+) | 3-cis-Hydroxy-b,e-Caroten-3'-one | 31.9963 | 551.4232 | Lipids and lipid-like molecules | 0.0000 | 0.0000 | 0.1782 | -1.6217 | -1.4435 |
| 15 | ESI (+) | 3-Hydroxyomeprazole | 37.2061 | 362.1165 | Organoheterocyclic compounds | 0.0007 | 0.0015 | -0.8589 | 14.7716 | 13.9127 |
| 16 | ESI (+) | 3-Indoleacetonitrile | 50.21295 | 157.0758 | Organoheterocyclic compounds | 0.0000 | 0.0000 | -0.5828 | -0.4598 | -1.0426 |
| 17 | ESI (+) | 4,8 Dimethylnonanoyl carnitine | 210.039 | 330.2632 | Lipids and lipid-like molecules | 0.0001 | 0.0002 | -0.1558 | -1.9358 | -2.0916 |
| 18 | ESI (+) | 4-Hydroxystachydrine | 312.785 | 160.0968 | Organic acids and derivatives | 0.0026 | 0.0045 | -1.5997 | 0.0977 | -1.5020 |
| 19 | ESI (+) | 5-Acetamidovalerate | 352.741 | 160.0968 | Lipids and lipid-like molecules | 0.0337 | 0.0479 | 0.2457 | -0.6164 | -0.3707 |
| 20 | ESI (+) | 5-Amino-3-oxohexanoate | 320.966 | 146.0810 | Organic acids and derivatives | 0.0000 | 0.0000 | 1.7904 | -2.7195 | -0.9291 |
| 21 | ESI (+) | 6-Chloro-N-(1-methylethyl)-1,3,5-triazine-2,4-diamine | 281.06 | 188.0704 | Organoheterocyclic compounds | 0.0000 | 0.0000 | -0.1917 | -0.8724 | -1.0641 |
| 22 | ESI (+) | alpha-Chaconine | 283.661 | 852.5219 | Lipids and lipid-like molecules | 0.0000 | 0.0000 | -0.1452 | 2.8864 | 2.7412 |
| 23 | ESI (+) | Avocadyne 4-acetate | 30.3055 | 327.2522 | Lipids and lipid-like molecules | 0.0003 | 0.0007 | -0.1345 | 6.0583 | 5.9238 |
| 24 | ESI (+) | Choline | 215.0235 | 524.3696 | Lipids and lipid-like molecules | 0.0000 | 0.0000 | -0.6418 | -0.8966 | -1.5384 |
| 25 | ESI (+) | Cryptoxanthin 5,6:5',8'-diepoxide | 33.4893 | 585.4275 | Lipids and lipid-like molecules | 0.0000 | 0.0000 | 0.4263 | -2.3780 | -1.9517 |
| 26 | ESI (+) | Cyclopassifloic acid A | 50.1886 | 537.3789 | Lipids and lipid-like molecules | 0.0000 | 0.0000 | 0.1337 | 3.3180 | 3.4518 |
| 27 | ESI (+) | Cyclovariegatin | 24.08155 | 355.0478 | Organoheterocyclic compounds | 0.0114 | 0.0178 | 1.5734 | -3.0832 | -1.5098 |
| 28 | ESI (+) | D-Alanine | 50.96325 | 175.0863 | Organoheterocyclic compounds | 0.0001 | 0.0001 | 1.0530 | 2.7634 | 3.8164 |
| 29 | ESI (+) | Demethylated antipyrine | 50.96325 | 175.0863 | Organoheterocyclic compounds | 0.0000 | 0.0000 | -0.4913 | -0.1594 | -0.6507 |
| 30 | ESI (+) | DG(18:1(11Z)/18:4(6Z,9Z,12Z,15Z)/0:0) | 30.2813 | 615.4975 | Lipids and lipid-like molecules | 0.0000 | 0.0000 | -0.6398 | -1.0382 | -1.6780 |
| 31 | ESI (+) | Diethanolamine | 333.549 | 106.0865 | Organic nitrogen compounds | 0.0049 | 0.0083 | 0.5520 | -0.7945 | -0.2425 |
| 32 | ESI (+) | Dodecanoylcarnitine | 210.012 | 344.2789 | Lipids and lipid-like molecules | 0.0006 | 0.0012 | 0.5624 | -0.8805 | -0.3182 |
| 33 | ESI (+) | Ecgonine | 331.81 | 186.1126 | Alkaloids and derivatives | 0.0000 | 0.0001 | -0.6626 | -0.4835 | -1.1460 |
| 34 | ESI (+) | Eujambin | 47.6102 | 659.1336 | Phenylpropanoids and polyketides | 0.0000 | 0.0000 | -0.6261 | -1.9254 | -2.5516 |
| 35 | ESI (+) | Glycerophosphocholine | 406.86 | 258.1099 | Lipids and lipid-like molecules | 0.0001 | 0.0001 | -1.4047 | -1.9521 | -3.3568 |
| 36 | ESI (+) | Hexadecanedioic acid mono-L-carnitine ester | 292.5575 | 430.3159 | Lipids and lipid-like molecules | 0.0000 | 0.0000 | 0.6662 | 0.8079 | 1.4741 |
| 37 | ESI (+) | Histamine | 49.6912 | 112.0758 | Organic nitrogen compounds | 0.0000 | 0.0000 | -0.2172 | -1.1599 | -1.3771 |
| 38 | ESI (+) | Homoarecoline | 361.319 | 170.1175 | Alkaloids and derivatives | 0.0003 | 0.0007 | -1.3553 | 0.1587 | -1.1966 |
| 39 | ESI (+) | Hypoxanthine | 231.4405 | 137.0457 | Organoheterocyclic compounds | 0.0001 | 0.0001 | 1.4712 | -3.3411 | -1.8699 |
| 40 | ESI (+) | Indole-3-methyl acetate | 277.3975 | 190.0862 | Organoheterocyclic compounds | 0.0007 | 0.0014 | -0.8296 | 0.4050 | -0.4246 |
| 41 | ESI (+) | Isobornyl propionate | 378.737 | 144.1020 | Lipids and lipid-like molecules | 0.0000 | 0.0001 | 0.6164 | -0.9876 | -0.3712 |
| 42 | ESI (+) | Isoleucyl-Isoleucine | 203.234 | 245.1857 | Organic acids and derivatives | 0.0000 | 0.0000 | -0.6360 | -1.0737 | -1.7097 |
| 43 | ESI (+) | L-Phenylalanine | 191.4105 | 279.1700 | Organic acids and derivatives | 0.0005 | 0.0011 | -0.6665 | 1.6870 | 1.0205 |
| 44 | ESI (+) | Lutein | 32.0091 | 568.4262 | Lipids and lipid-like molecules | 0.0000 | 0.0000 | 0.0691 | -1.0928 | -1.0236 |
| 45 | ESI (+) | LysoPC(14:0/0:0) | 223.666 | 468.3075 | Lipids and lipid-like molecules | 0.0000 | 0.0000 | -0.3539 | -1.8455 | -2.1993 |
| 46 | ESI (+) | LysoPC(16:0) | 219.245 | 496.3394 | Lipids and lipid-like molecules | 0.0000 | 0.0000 | -0.2133 | -1.0089 | -1.2222 |
| 47 | ESI (+) | LysoPC(18:3(6Z,9Z,12Z)) | 219.861 | 518.3223 | Lipids and lipid-like molecules | 0.0000 | 0.0000 | -0.2133 | -1.0089 | -1.2222 |
| 48 | ESI (+) | LysoPC(22:2(13Z,16Z)) | 54.54955 | 576.3953 | Lipids and lipid-like molecules | 0.0000 | 0.0000 | 0.1243 | 1.1778 | 1.3021 |
| 49 | ESI (+) | LysoPC(24:0) | 208.632 | 608.4655 | Lipids and lipid-like molecules | 0.0000 | 0.0000 | 0.0176 | -1.3619 | -1.3444 |
| 50 | ESI (+) | LysoPC(P-16:0) | 211.266 | 480.3442 | Lipids and lipid-like molecules | 0.0000 | 0.0000 | -0.0365 | -1.9615 | -1.9981 |
| 51 | ESI (+) | LysoPE(18:1(9Z)/0:0) | 222.5545 | 480.3081 | Lipids and lipid-like molecules | 0.0000 | 0.0000 | -0.5576 | -0.6446 | -1.2022 |
| 52 | ESI (+) | PC(22:5(7Z,10Z,13Z,16Z,19Z)/18:3(6Z,9Z,12Z)) | 53.6755 | 830.5741 | Lipids and lipid-like molecules | 0.0000 | 0.0000 | 0.0204 | -1.1730 | -1.1526 |
| 53 | ESI (+) | PE(22:2(13Z,16Z)/14:1(9Z)) | 170.087 | 742.5390 | Lipids and lipid-like molecules | 0.0000 | 0.0001 | -0.5890 | -0.4253 | -1.0143 |
| 54 | ESI (+) | PE(22:6(4Z,7Z,10Z,13Z,16Z,19Z)/22:6(4Z,7Z,10Z,13Z,16Z,19Z)) | 285.4695 | 836.5276 | Lipids and lipid-like molecules | 0.0000 | 0.0000 | -0.4749 | -1.6583 | -2.1332 |
| 55 | ESI (+) | PE(P-18:1(11Z)/18:2(9Z,12Z)) | 161.508 | 726.5411 | Lipids and lipid-like molecules | 0.0000 | 0.0000 | 0.6538 | -1.7522 | -1.0984 |
| 56 | ESI (+) | PE(P-18:1(11Z)/18:3(6Z,9Z,12Z)) | 158.897 | 724.5284 | Lipids and lipid-like molecules | 0.0000 | 0.0000 | 0.5229 | -1.2951 | -0.7722 |
| 57 | ESI (+) | PE(P-18:1(9Z)/16:1(9Z)) | 165.007 | 700.5280 | Lipids and lipid-like molecules | 0.0000 | 0.0000 | 1.1492 | -2.2530 | -1.1038 |
| 58 | ESI (+) | PE(P-18:1(9Z)/20:3(5Z,8Z,11Z)) | 156.389 | 752.5587 | Lipids and lipid-like molecules | 0.0000 | 0.0000 | 0.5608 | -1.4065 | -0.8456 |
| 59 | ESI (+) | Proline betaine | 290.085 | 144.1018 | Organic acids and derivatives | 0.0098 | 0.0156 | -0.5933 | -1.0669 | -1.6602 |
| 60 | ESI (+) | PS(15:0/18:0) | 49.3019 | 750.5213 | Lipids and lipid-like molecules | 0.0000 | 0.0000 | 0.1208 | 2.3076 | 2.4284 |
| 61 | ESI (+) | SM(d17:1/24:1(15Z)) | 199.964 | 799.6699 | Organic nitrogen compounds | 0.0000 | 0.0000 | 0.2316 | -0.8484 | -0.6168 |
| 62 | ESI (+) | Sorbitol | 310.221 | 221.0420 | Organic oxygen compounds | 0.0000 | 0.0000 | 0.4384 | 5.4909 | 5.9293 |
| 63 | ESI (+) | Sphinganine | 58.8441 | 302.3047 | Organic nitrogen compounds | 0.0002 | 0.0004 | -0.7830 | 1.4069 | 0.6239 |
| 64 | ESI (+) | Sphingosine | 87.7463 | 300.2890 | Organic nitrogen compounds | 0.0000 | 0.0000 | -0.0093 | -2.1909 | -2.2002 |
| 65 | ESI (+) | Synephrine acetonide | 214.684 | 208.1330 | Benzenoids | 0.0022 | 0.0040 | -0.5129 | -2.9610 | -3.4739 |
| 66 | ESI (+) | Tripropylamine | 139.706 | 144.1745 | Organonitrogen compounds | 0.0000 | 0.0000 | -0.0124 | -13.0576 | -13.0700 |
| 67 | ESI (+) | Uracil | 171.008 | 113.0347 | Organoheterocyclic compounds | 0.0000 | 0.0000 | -1.0191 | -0.4780 | -1.4971 |
| 68 | ESI (-) | (R)-3-Hydroxy-tetradecanoic acid | 60.7456 | 243.1970 | Lipids and lipid-like molecules | 0.0000 | 0.0000 | 0.6186 | -1.2547 | -0.6361 |
| 69 | ESI (-) | [12]-Gingerol | 153.352 | 377.2709 | Benzenoids | 0.0000 | 0.0000 | 0.1250 | -1.3089 | -1.1840 |
| 70 | ESI (-) | 1,3,5-Trihydroxybenzene | 318.713 | 125.0240 | Benzenoids | 0.0000 | 0.0000 | 0.1836 | 1.0625 | 1.2461 |
| 71 | ESI (-) | 16(17)-EpDPE | 49.90875 | 343.2288 | Lipids and lipid-like molecules | 0.0000 | 0.0000 | -1.8562 | 0.3211 | -1.5351 |
| 72 | ESI (-) | 16-Hydroxy hexadecanoic acid | 51.5429 | 271.2282 | Lipids and lipid-like molecules | 0.0000 | 0.0000 | 0.6204 | -0.9758 | -0.3554 |
| 73 | ESI (-) | 1-deoxy-1-(N6-lysino)-D-fructose | 316.969 | 132.0298 | Organic acids and derivatives | 0.0279 | 0.0471 | -0.6508 | 0.0173 | -0.6335 |
| 74 | ESI (-) | 1H-Indole-2,3-dione | 46.3076 | 146.0244 | Organoheterocyclic compounds | 0.0430 | 0.0686 | -0.5425 | 0.3104 | -0.2321 |
| 75 | ESI (-) | 1H-Indole-3-carboxaldehyde | 39.97085 | 144.0451 | Organoheterocyclic compounds | 0.0000 | 0.0000 | -0.1188 | -0.9973 | -1.1161 |
| 76 | ESI (-) | 2',4',6'-Trihydroxyacetophenone | 45.4896 | 167.0348 | Benzenoids | 0.0001 | 0.0003 | 1.7281 | -2.3692 | -0.6410 |
| 77 | ESI (-) | 24-Epibrassinolide | 177.871 | 479.3398 | Lipids and lipid-like molecules | 0.0000 | 0.0000 | 0.0598 | -1.1466 | -1.0867 |
| 78 | ESI (-) | 2-acetyl-1-alkyl-sn-glycero-3-phosphocholine | 215.309 | 522.3568 | Lipids and lipid-like molecules | 0.0000 | 0.0000 | -0.0933 | -1.4992 | -1.5925 |
| 79 | ESI (-) | 2-Pyrocatechuic acid | 24.5268 | 153.0191 | Benzenoids | 0.0000 | 0.0000 | 2.4435 | -4.3085 | -1.8650 |
| 80 | ESI (-) | 3b-Hydroxy-5-cholenoic acid | 60.12495 | 373.2761 | Lipids and lipid-like molecules | 0.0000 | 0.0001 | -0.8775 | -0.7852 | -1.6627 |
| 81 | ESI (-) | 3-Hydroxycapric acid | 61.0665 | 187.1340 | Organic acids and derivatives | 0.0000 | 0.0000 | 0.4402 | -0.9938 | -0.5536 |
| 82 | ESI (-) | 5-Methylcytidine | 214.6655 | 256.0942 | Nucleosides, nucleotides, and analogues | 0.0000 | 0.0000 | -1.2475 | 4.3753 | 3.1277 |
| 83 | ESI (-) | 9,10-DHOME | 71.8115 | 313.2390 | Lipids and lipid-like molecules | 0.0296 | 0.0494 | 1.1128 | -0.4217 | 0.6911 |
| 84 | ESI (-) | 9-Decenoic acid | 51.5541 | 169.1231 | Lipids and lipid-like molecules | 0.0001 | 0.0004 | 0.4294 | -0.8816 | -0.4522 |
| 85 | ESI (-) | Acrylic acid | 220.5615 | 186.0560 | Organoheterocyclic compounds | 0.0032 | 0.0071 | 0.4181 | 1.1115 | 1.5296 |
| 86 | ESI (-) | Aldehydo-D-xylose | 150.6795 | 149.0452 | Organic oxygen compounds | 0.0000 | 0.0000 | 0.0198 | 1.2851 | 1.3048 |
| 87 | ESI (-) | Allose | 109.268 | 179.0559 | Organic oxygen compounds | 0.0000 | 0.0001 | 1.0176 | 2.3376 | 3.3552 |
| 88 | ESI (-) | Alpha-Linolenic acid | 43.80825 | 277.2177 | Lipids and lipid-like molecules | 0.0000 | 0.0001 | 0.7296 | -1.3852 | -0.6556 |
| 89 | ESI (-) | Androsterone sulfate | 28.1762 | 369.1744 | Lipids and lipid-like molecules | 0.0056 | 0.0116 | 0.6441 | -0.7411 | -0.0970 |
| 90 | ESI (-) | Ascorbic acid | 90.6637 | 175.0246 | Organoheterocyclic compounds | 0.0000 | 0.0000 | 0.1530 | 0.7302 | 0.8832 |
| 91 | ESI (-) | Benzenebutanoic acid | 67.38095 | 163.0763 | Benzenoids | 0.0000 | 0.0000 | 0.6314 | -2.7930 | -2.1616 |
| 92 | ESI (-) | beta-Alanine | 387.05 | 88.0399 | Organic acids and derivatives | 0.0001 | 0.0003 | 0.0629 | -0.6129 | -0.5500 |
| 93 | ESI (-) | bicyclo-PGE2 | 93.9737 | 333.2082 | Lipids and lipid-like molecules | 0.0000 | 0.0000 | 0.1917 | -1.8682 | -1.6765 |
| 94 | ESI (-) | But-2-enoic acid | 77.8467 | 85.0289 | Lipids and lipid-like molecules | 0.0000 | 0.0000 | 0.2387 | 0.8829 | 1.1216 |
| 95 | ESI (-) | cis-Vaccenic acid | 41.8594 | 281.2490 | Lipids and lipid-like molecules | 0.0243 | 0.0417 | 0.5460 | -0.3888 | 0.1572 |
| 96 | ESI (-) | Cytidine | 407.2365 | 242.0801 | Nucleosides, nucleotides, and analogues | 0.0005 | 0.0013 | -0.6798 | -1.7250 | -2.4048 |
| 97 | ESI (-) | Dehydroepiandrosterone sulfate | 27.2549 | 367.1593 | Lipids and lipid-like molecules | 0.0015 | 0.0037 | 0.5016 | -0.7569 | -0.2553 |
| 98 | ESI (-) | D-Glucose | 231.078 | 179.0559 | Organic oxygen compounds | 0.0000 | 0.0000 | -0.2254 | -0.3526 | -0.5781 |
| 99 | ESI (-) | D-Glutamine | 391.436 | 145.0613 | Organic acids and derivatives | 0.0290 | 0.0486 | 1.0514 | -0.4963 | 0.5551 |
| 100 | ESI (-) | Dihydrolipoate | 49.0004 | 207.0512 | Lipids and lipid-like molecules | 0.0000 | 0.0000 | 0.2465 | 1.2205 | 1.4670 |
| 101 | ESI (-) | D-Mannose | 295.764 | 179.0561 | Organic oxygen compounds | 0.0000 | 0.0000 | -0.3485 | 1.4795 | 1.1311 |
| 102 | ESI (-) | Dopamine | 155.028 | 152.0714 | Benzenoids | 0.0006 | 0.0015 | 2.9194 | 3.6546 | 6.5740 |
| 103 | ESI (-) | D-Ribose | 77.83575 | 149.0452 | Organic oxygen compounds | 0.0000 | 0.0000 | 0.3269 | 1.4578 | 1.7847 |
| 104 | ESI (-) | dUMP | 231.079 | 307.0353 | Nucleosides, nucleotides, and analogues | 0.0000 | 0.0000 | -0.2391 | -0.3675 | -0.6066 |
| 105 | ESI (-) | Eicosadienoic acid | 41.8164 | 307.2645 | Lipids and lipid-like molecules | 0.0452 | 0.0717 | 0.6142 | -0.4917 | 0.1225 |
| 106 | ESI (-) | Eicosapentaenoic acid | 42.3938 | 301.2179 | Lipids and lipid-like molecules | 0.0001 | 0.0002 | -0.3915 | -1.4841 | -1.8757 |
| 107 | ESI (-) | Estrone glucuronide | 48.722 | 445.1908 | Lipids and lipid-like molecules | 0.0000 | 0.0001 | 0.1494 | 1.9024 | 2.0517 |
| 108 | ESI (-) | Ethyl dodecanoate | 46.2341 | 227.2017 | Lipids and lipid-like molecules | 0.0002 | 0.0005 | 0.6677 | -1.0322 | -0.3645 |
| 109 | ESI (-) | Ethyl hexadecanoate | 41.8415 | 283.2642 | Lipids and lipid-like molecules | 0.0122 | 0.0230 | 0.5305 | -0.4138 | 0.1167 |
| 110 | ESI (-) | Formylanthranilic acid | 76.4668 | 164.0352 | Benzenoids | 0.0001 | 0.0004 | -0.1026 | -1.1970 | -1.2996 |
| 111 | ESI (-) | Galacturonic acid | 308.554 | 193.0353 | Organic oxygen compounds | 0.0000 | 0.0000 | 0.0188 | 1.4820 | 1.5008 |
| 112 | ESI (-) | gamma-CEHC | 78.50615 | 263.1294 | Organoheterocyclic compounds | 0.0000 | 0.0000 | 0.7266 | -1.4443 | -0.7177 |
| 113 | ESI (-) | Glutaric acid | 198.628 | 161.0452 | Lipids and lipid-like molecules | 0.0003 | 0.0007 | -0.0500 | 1.0358 | 0.9858 |
| 114 | ESI (-) | Glycine | 382.504 | 74.0241 | Organic acids and derivatives | 0.0014 | 0.0034 | -0.3636 | -0.5666 | -0.9302 |
| 115 | ESI (-) | Glycyl-glycine | 369.865 | 131.0458 | Organic acids and derivatives | 0.0047 | 0.0098 | -0.6385 | 0.6123 | -0.0263 |
| 116 | ESI (-) | Heptadecanoic acid | 42.8274 | 269.2488 | Lipids and lipid-like molecules | 0.0003 | 0.0009 | 0.5755 | -0.9894 | -0.4139 |
| 117 | ESI (-) | Hexylresorcinol | 50.4754 | 193.1233 | Benzenoids | 0.0000 | 0.0000 | 0.8626 | -1.8958 | -1.0332 |
| 118 | ESI (-) | Hippuric acid | 204.134 | 178.0508 | Benzenoids | 0.0000 | 0.0000 | 1.6399 | -3.1979 | -1.5579 |
| 119 | ESI (-) | Hydrogen phosphate | 165.972 | 96.9692 | Homogeneous non-metal compounds | 0.0002 | 0.0006 | 0.3032 | -0.3789 | -0.0757 |
| 120 | ESI (-) | Hypogeic acid | 44.1806 | 253.2177 | Lipids and lipid-like molecules | 0.0068 | 0.0136 | 0.6196 | -0.5342 | 0.0854 |
| 121 | ESI (-) | Hypoxanthine | 177.494 | 135.0307 | Organoheterocyclic compounds | 0.0001 | 0.0002 | -0.3321 | -1.1420 | -1.4741 |
| 122 | ESI (-) | Imidazoleacetic acid | 93.7819 | 125.0352 | Organoheterocyclic compounds | 0.0000 | 0.0000 | -0.0877 | -0.5850 | -0.6727 |
| 123 | ESI (-) | Inosine | 231.087 | 267.0738 | Nucleosides, nucleotides, and analogues | 0.0001 | 0.0002 | 1.4031 | -2.9642 | -1.5610 |
| 124 | ESI (-) | L-Glutamic acid | 70.9111 | 146.0456 | Organic acids and derivatives | 0.0000 | 0.0000 | 0.4007 | 0.5978 | 0.9986 |
| 125 | ESI (-) | L-Gulonolactone | 197.374 | 177.0403 | Organoheterocyclic compounds | 0.0000 | 0.0000 | 0.0092 | 0.7715 | 0.7807 |
| 126 | ESI (-) | Linoelaidic acid | 42.1753 | 279.2332 | Lipids and lipid-like molecules | 0.0004 | 0.0010 | 0.2869 | -0.6639 | -0.3770 |
| 127 | ESI (-) | LysoPA(16:0/0:0) | 214.149 | 409.2367 | Lipids and lipid-like molecules | 0.0000 | 0.0000 | -0.0867 | -1.4839 | -1.5706 |
| 128 | ESI (-) | LysoPA(18:1(9Z)/0:0) | 217.959 | 435.2526 | Lipids and lipid-like molecules | 0.0000 | 0.0000 | -0.1423 | -0.9021 | -1.0444 |
| 129 | ESI (-) | m-Coumaric acid | 93.6909 | 163.0399 | Phenylpropanoids and polyketides | 0.0079 | 0.0156 | -0.4979 | 0.5408 | 0.0429 |
| 130 | ESI (-) | Mesylate | 111.662 | 94.9802 | Organic compounds | 0.0001 | 0.0003 | 0.5091 | -0.9826 | -0.4735 |
| 131 | ESI (-) | Methyl jasmonate | 169.05 | 223.1342 | Lipids and lipid-like molecules | 0.0000 | 0.0000 | 0.4309 | -1.6726 | -1.2417 |
| 132 | ESI (-) | N-Acetylarylamine | 204.646 | 134.0607 | Benzenoids | 0.0000 | 0.0001 | 1.5491 | -3.1189 | -1.5698 |
| 133 | ESI (-) | N-Acetyl-L-methionine | 210.7755 | 190.0542 | Organic acids and derivatives | 0.0008 | 0.0020 | -0.8706 | 0.0379 | -0.8327 |
| 134 | ESI (-) | N-Acetylserine | 309.8565 | 146.0455 | Organic acids and derivatives | 0.0000 | 0.0000 | -0.0429 | 0.6632 | 0.6203 |
| 135 | ESI (-) | Oleic acid | 93.64465 | 281.2491 | Lipids and lipid-like molecules | 0.0022 | 0.0050 | 0.5493 | -1.1273 | -0.5780 |
| 136 | ESI (-) | Oxoadipic acid | 146.274 | 159.0296 | Organic acids and derivatives | 0.0000 | 0.0000 | 0.2829 | 0.8128 | 1.0957 |
| 137 | ESI (-) | Palmitic acid | 44.117 | 255.2330 | Lipids and lipid-like molecules | 0.0028 | 0.0063 | 0.3954 | -0.5332 | -0.1378 |
| 138 | ESI (-) | Pantothenic acid | 282.5055 | 218.1034 | Organooxygen compounds | 0.0003 | 0.0007 | -0.3524 | -0.2736 | -0.6260 |
| 139 | ESI (-) | Parabanic Acid | 142.789 | 145.0503 | Lipids and lipid-like molecules | 0.6034 | 0.6573 | 0.1998 | -0.2449 | -0.0451 |
| 140 | ESI (-) | Pentadecanoic acid | 45.1191 | 241.2177 | Lipids and lipid-like molecules | 0.0000 | 0.0001 | 0.0068 | -0.8136 | -0.8068 |
| 141 | ESI (-) | Perillic acid | 60.80785 | 165.0919 | Lipids and lipid-like molecules | 0.0000 | 0.0000 | 0.4494 | -2.0405 | -1.5910 |
| 142 | ESI (-) | Pyrocatechol | 22.7459 | 109.0289 | Benzenoids | 0.0000 | 0.0000 | 2.4650 | -4.8980 | -2.4330 |
| 143 | ESI (-) | Pyruvic acid | 231.08 | 87.0083 | Organic acids and derivatives | 0.0000 | 0.0001 | -0.1170 | -0.2946 | -0.4116 |
| 144 | ESI (-) | Rhamnose | 209.237 | 163.0609 | Organic oxygen compounds | 0.0000 | 0.0001 | 0.9026 | 0.0107 | 0.9134 |
| 145 | ESI (-) | Ribitol | 97.13225 | 151.0610 | Organic oxygen compounds | 0.0098 | 0.0188 | 0.6434 | -0.8703 | -0.2269 |
| 146 | ESI (-) | Sarcosine | 364.814 | 88.0398 | Organic acids and derivatives | 0.0000 | 0.0000 | 0.0674 | -0.5782 | -0.5107 |
| 147 | ESI (-) | Sorbitol | 309.7235 | 181.0715 | Organic oxygen compounds | 0.0000 | 0.0001 | 1.6765 | 9.3862 | 11.0627 |
| 148 | ESI (-) | Stearic acid | 50.6092 | 299.2599 | Lipids and lipid-like molecules | 0.0000 | 0.0000 | 0.0342 | -0.8229 | -0.7887 |
| 149 | ESI (-) | Succinic acid | 397.46 | 117.0189 | Organic acids and derivatives | 0.0000 | 0.0000 | -0.4891 | -0.4659 | -0.9550 |
| 150 | ESI (-) | Succinic acid semialdehyde | 248.972 | 101.0239 | Lipids and lipid-like molecules | 0.0000 | 0.0000 | -1.7095 | -0.5056 | -2.2152 |
| 151 | ESI (-) | Terephthalic acid | 365.433 | 165.0190 | Benzenoids | 0.0011 | 0.0027 | 0.0926 | -0.0955 | -0.0029 |
| 152 | ESI (-) | Tetradecanedioic acid | 241.849 | 257.1762 | Lipids and lipid-like molecules | 0.0011 | 0.0026 | 0.5547 | -1.8844 | -1.3297 |
| 153 | ESI (-) | Theophylline | 56.33325 | 179.0574 | Organoheterocyclic compounds | 0.0001 | 0.0004 | 2.9023 | -3.5031 | -0.6009 |
| 154 | ESI (-) | Tridecanoic acid | 35.6881 | 213.1862 | Lipids and lipid-like molecules | 0.0005 | 0.0014 | -0.6084 | -0.1590 | -0.7675 |
| 155 | ESI (-) | Uracil | 168.292 | 111.0307 | Organoheterocyclic compounds | 0.0000 | 0.0000 | -0.6311 | -0.4406 | -1.0718 |
| 156 | ESI (-) | Uridine | 104.8235 | 227.0675 | Nucleosides, nucleotides, and analogues | 0.0001 | 0.0002 | -0.7646 | -0.4468 | -1.2113 |
